# Mapping cellular targets of covalent cancer drugs in the entire mammalian body

**DOI:** 10.1016/j.cell.2025.11.030

**Published:** 2025-12-22

**Authors:** Zhengyuan Pang, Verina H. Leung, Cailynn C. Wang, Ahmadreza Attarpour, Anthony Rinaldi, Hanbing Shen, Maria Dolores Moya-Garzon, Logan H. Sigua, Claire Rammel, Alexandra Selke, Christopher Glynn, Melaina Yender, Senhan Xu, Javid J. Moslehi, Peng Wu, Jonathan Z. Long, Maged Goubran, Benjamin F. Cravatt, Li Ye

**Affiliations:** 1 Department of Neuroscience, Dorris Neuroscience Center, Scripps Research, San Diego, CA, USA; 2 Howard Hughes Medical Institute, Chevy Chase, MD, USA; 3 Department of Chemistry, The Scripps Research Institute, La Jolla, CA 92037, USA; 4 Physical Sciences Platform, Sunnybrook Research Institute, Toronto, ON, Canada; 5 Department of Medical Biophysics, University of Toronto, Toronto, ON, Canada; 6 Department of Pathology, Stanford School of Medicine, Stanford, CA, USA; 7 Sarafan ChEM-H, Stanford University, Stanford, CA, USA; 8 Section of Cardio-Oncology & Immunology, Cardiovascular Research Institute (CVRI), University of California, San Francisco, School of Medicine, San Francisco, CA 94158, USA; 9 Department of Molecular and Cellular Biology, The Scripps Research Institute, La Jolla, CA 92037, USA; 10 Hurvitz Brain Sciences, Sunnybrook Health Sciences Centre, Toronto, ON, Canada

## Abstract

As our understanding of biological systems reaches single-cell and high spatial resolutions, it becomes imperative that pharmacological approaches match this precision to understand drug actions. This need is particularly urgent for the targeted covalent inhibitors that are currently re-entering the stage for cancer treatments. By leveraging the unique kinetics of click reactions, we developed volumetric clearing-assisted tissue click chemistry (vCATCH) to enable deep and homogeneous click labeling across the three-dimensional (3D) mammalian body. With simple and passive incubation steps, vCATCH offers cellular-resolution drug imaging in the entire adult mouse. We combined vCATCH with hydrogel-based reinforcement of three-dimensional imaging solvent-cleared organs (HYBRiD) imaging and virtual reality to visualize and quantify *in vivo* targets of two clinical cancer drugs, afatinib and ibrutinib, which recapitulated their known pharmacological distribution and revealed previously unreported tissue and cell-type engagement potentially linked to off-target effects. vCATCH provides a body-wide, unbiased platform to map covalent drug engagements at unprecedented scale and precision.

## In brief

vCATCH is a volumetric tissue labeling technique enabling cellular-resolution mapping of covalent drug targets throughout the entire mammalian body.

## Graphical Abstract

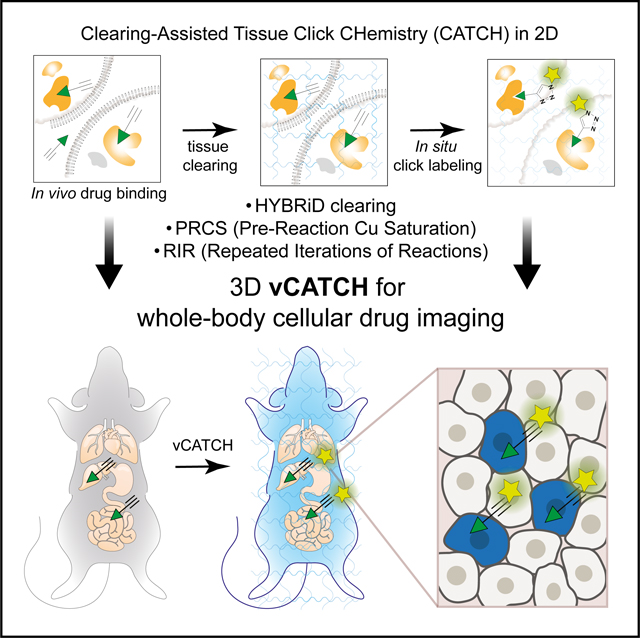

## INTRODUCTION

The primary binding sites for drug molecules are critical determinants of drug efficacy and toxicity. Although molecular target identification strategies, such as chemoproteomics,^1–5^ chemogenomics,^6–9^ and thermal shift profiling,^10–13^ have revolutionized our understanding of drug activities, mapping *in vivo* drug targets with spatial and cellular resolution remains challenging. This shortfall is even more pressing in light of the innovations in genomics and imaging techniques over the past decade that have enabled the characterization of tissue architecture at the single-cell level with spatial resolution.^14–20^ This has led to a widening gap between our understanding of native biological systems and our ability to track *in vivo* drug actions within these systems, as the latter still primarily rely on low-resolution methods such as radioactive imaging. It is imperative that we develop methods to visualize *in situ* drug engagement with cellular resolution across tissue compartments and organ systems to better understand *in vivo* drug actions.

One promising avenue for imaging drug targets is fluorescence imaging, which has been widely used for visualizing endogenous biomolecules like DNA, mRNA, and proteins. However, directly imaging drug molecules conjugated with a fluorescent tag is often not suitable, as a larger tag could distort drug activity and distribution *in vivo*. As an alternative approach, we recently developed a method to visualize drug molecules in tissue sections by combining tissue clearing with *in situ* click chemistry (CATCH).^21^ However, efforts to extend CATCH from thin sections to large, heterogeneous three-dimensional (3D) tissue environments face significant challenges similar to those typically encountered by whole-mount immunostaining or *in situ* hybridization methods, such as the accumulation of probes at the tissue surface, uneven staining across the tissue depth, and insufficient labeling at the center of large samples.^22–26^

Here, we develop new CATCH capacities to enable rapid and homogeneous *in situ* click labeling in ultra-large 3D tissues at the scale of a whole mouse body. This new technology leverages the unique characteristics of click chemistry by introducing pre-reaction copper saturation (PRCS) and repeated iterations of reaction (RIR). The resulting volumetric CATCH (vCATCH) method, when combined with hydrogel-based reinforcement of three-dimensional imaging solvent-cleared organs (DISCO) (HYBRiD) clearing,^27^ is further highlighted by its marked simplicity, as it only relies on easy and passive incubation steps to achieve whole-body labeling and imaging.

## RESULTS

### Establishing the principles for deep tissue click reactions

To achieve 3D click labeling, we turned to recent advances in whole-brain and whole-body immunostaining techniques.^24,28–35^ As summarized by previous works,^36–39^ two main strategies have been used to achieve deep tissue antibody penetration: (1) enhancing diffusion of the probe (e.g., antibody) into the tissue and (2) minimizing probe-target interactions (e.g., antibody-antigen binding) during the diffusion stage while maximizing reaction efficiency at the staining stage, such that the large difference in reaction rates between the two stages will prevent probe depletion at the tissue surface and facilitate homogeneous tissue labeling.^23,33,34^ Biophysics studies have demonstrated that changing the pH, salt concentration, and other physicochemical parameters can modulate antibody-antigen binding kinetics by 4- to 400-fold.^40,41^ We hypothesized that we could adopt a similar strategy to enable 3D click reactions. CATCH relies on copper-catalyzed alkyne azide cycloaddition (CuAAC) to label target-bound drug molecules with fluorescent signals. As its name indicates, the kinetics of CuAAC strictly depend on the catalytic Cu(I)-ligand complex, but for ease of handling, a Cu(II) compound, typically CuSO_4_, is used during the diffusion stage and then a reducing agent (e.g., sodium ascorbate) is added *in situ* to generate Cu(I) at the binding stage. Compared with uncatalyzed conditions, the generation of catalytic Cu(I) can lower the energy barrier by 14.9–18.4 kcal/mol, or an equivalent of a 7–8 orders of magnitude increase, favoring the cycloaddition that attaches the fluorescent probe to the drug *in situ*.^42^ Thus, by temporarily preventing the generation of Cu(I) from Cu(II) through withholding the reducing agent sodium ascorbate, in principle, we could modulate the probe-target reaction by 10^7–8^-fold between the diffusion and binding stages, which could improve the penetration depth of CATCH in large tissues.

**Figure 1.**
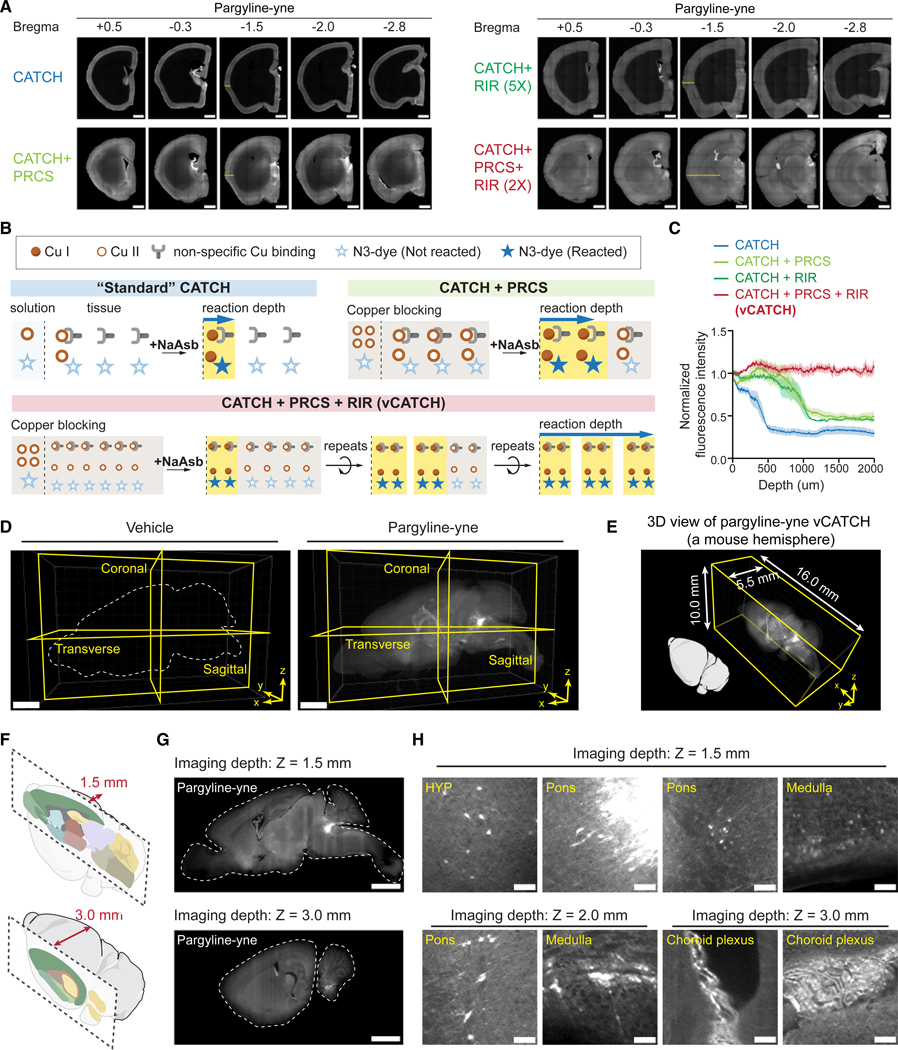
Establishing the principles for deep tissue click reactions (A) Brain-wide characterization of click labeling in pargyline-yne-treated hemispheres (10 mg/kg, 1 h i.p.). PRCS and RIRs (1 h per iteration) enhanced labeling depth and generated homogeneous click labeling across the whole hemisphere. (B) Schematics of copper competition in tissue. Copper binding sites could chelate copper and limit the available free copper for CuAAC catalyst conversion, thereby impairing click labeling efficiency deep in tissues. (C) Quantification of click labeling depth along the marked lines in (A). Four hemispheres in each condition. (D) Representative 3D rendering of vehicle and pargyline-yne-treated hemispheres. (E) 3D rendering of pargyline-yne-treated hemispheres showing the total imaging volume. (F) Schematics of imaging depth shown in (G), highlighting changes in different brain regions covered. (G) Sagittal 2D views of pargyline-yne-treated hemispheres at imaging depths Z = 1.5 and 3.0 mm as indicated in (F). (H) Zoomed-in views showing pargyline-yne-enriched neurons and brain regions across the hemisphere. Data are plotted as mean ± SEM. Scale bars: 1,000 μm (A), 2,000 μm (D and G), and 100 μm (H). See also Figure S1.

For method development, we used the covalent monoamine oxidase (MAO) inhibitor pargyline, whose alkyne probe pargyline-yne has previously been characterized by activity-based protein profiling (ABPP) and 2D-CATCH studies (Figure S1A).^21,43^ Although CATCH has demonstrated homogeneous click labeling of N_3_-dye in 500 μm tissues,^21^ when tested in mouse hemispheres, even with the aforementioned catalyst withholding strategy, only the surface of the mouse brain was labeled with a depth of 0.4 mm (Figure 1A). First, we used mouse brains treated with the fatty acid amide hydrolase (FAAH) inhibitor PF7845-yne (Figure S1A), another CATCH probe we previously established, to determine whether other CATCH components might have trouble diffusing through the tissue.^21,44^ We removed individual reagents from the diffusion step and evaluated the resulting labeling depth. For instance, eliminating the N_3_-dye, the largest molecular weight component, in the diffusion step did not affect labeling depth, suggesting N_3_-dye diffusion is not a limiting factor (Figures S1B and S1C). By contrast, removing CuSO_4_ or ligand strongly reduced labeling depth in these conditions, suggesting that the catalyst is still likely the diffusion bottleneck (Figures S1B and S1C).

Cu is well known to bind multiple amino acid side chains (i.e., cysteine and histidine).^45–48^ We therefore hypothesized that the ubiquitous copper binding sites could consume free Cu(II) as it diffuses into the sample, resulting in insufficient catalyst conversion in deeper tissues (Figure 1B). Unlike typical staining, in which increasing dye or antibody concentrations tends to result in nonspecific labeling and high background, high Cu(II) alone, as a catalytic precursor, should not lead to nonspecific labeling. Based on this theoretical prediction, we reasoned that excessive Cu(II) could be used to pre-saturate potential copper binding sites (therefore “blocking” them) to boost catalytic activity in deeper tissues. Importantly, this strategy should not increase nonspecific labeling or lead to high background. Indeed, we observed that incorporating a 10× Cu excess PRCS step increased the reaction depth to 1 mm while maintaining the signal-to-noise ratio after a single 1-h reaction (Figures 1A–1C).

We next sought to modify CATCH to reach targets in tissues deeper than 1 mm. Although increasing reaction time could potentially increase labeling depth, prolonged reactions would deplete the reducing agent in the buffer, which could eventually lead to reactive oxygen species (ROS) buildup and side reactions.^49–51^ Instead, we refreshed the click reaction cocktail every hour, a procedure we termed RIRs. In practice, PRCS plus two rounds of RIR resulted in full-depth, homogeneous CATCH labeling across the mouse hemisphere (Figures 1A–1C; Video S1). We term this approach vCATCH.

### vCATCH quantitatively profiles brain-wide targets of pargyline

After achieving brain-wide labeling, we employed vCATCH to unbiasedly map drug targets of pargyline. After intraperitoneal (i.p.) injection of pargyline-yne (10 mg/kg) or vehicle (non-drug treated) in wild-type mice, mouse hemispheres were harvested and cleared by HYBRiD. Hemispheres then underwent 1 day of PRCS and two rounds of RIR before imaging with lightsheet fluorescence microscopy (Figure 1D). Importantly, vCATCH allowed the use of high-throughput, paralleled passive processing to generate cohort-scale pargyline-labeled brains with marked reproducibility, critical for performing biologically meaningful analysis of its cellular targets across the whole brain (Figures 1E–1H, S1D, and S1E).

First, we manually verified pargyline probe binding in the brain based on previously reported regional targets.^21^ These regions included distinct cellular structures in the pons and hypothalamus (HYP) (Figures 1H and S1E). In addition, we found pargyline-yne-enriched cells in the choroid plexus (in the ventricle) and the medulla (Figures 1H and S1E). We also observed additional neuron-like structures in the midbrain (Figure S1E). These findings demonstrated that vCATCH maintained high fidelity in large tissue volumes and across the whole brain.

**Figure 2.**
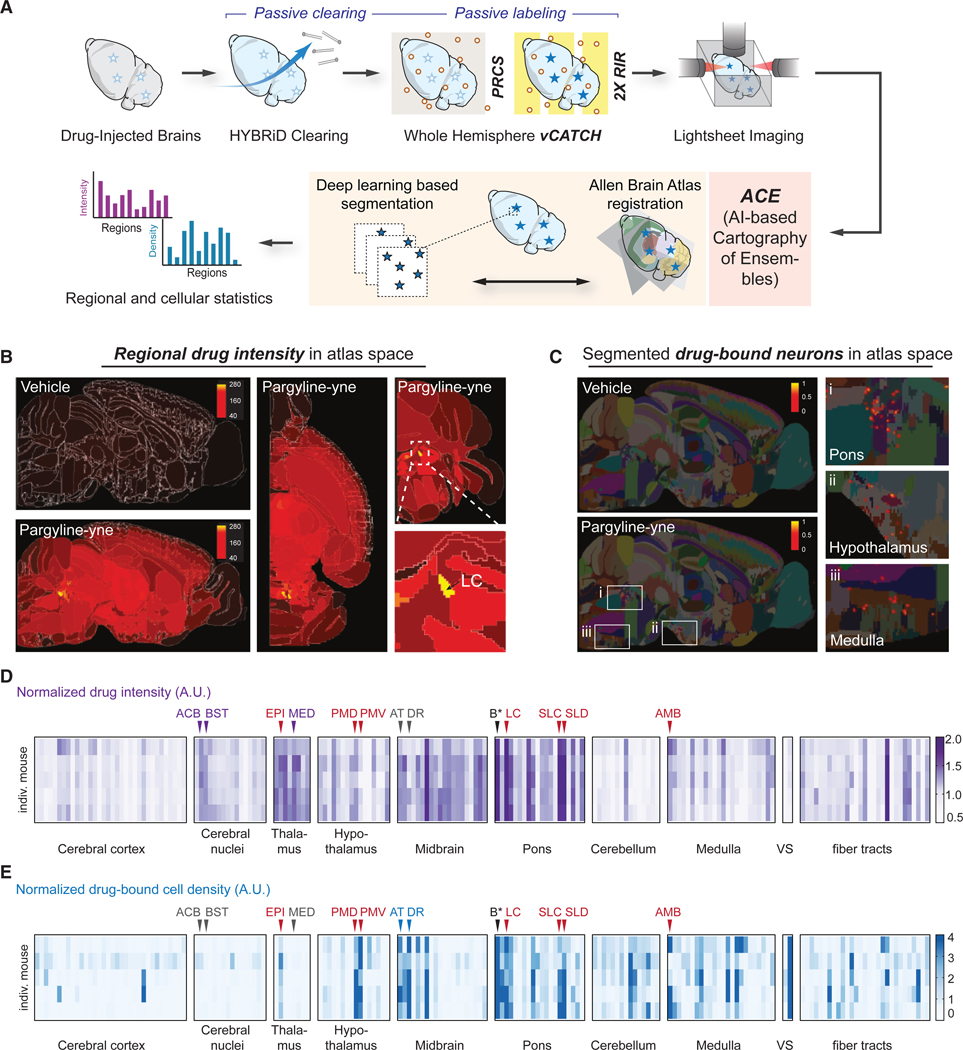
ACE analysis of pargyline-yne targets across the hemisphere (A) Schematics of the ACE analysis pipeline for brain-wide pargyline-yne (10 mg/kg, 1 h i.p.) target identification. (B) Representative regional drug intensity quantification in atlas space. A white boundary indicates brain regions by the ABA. The LC showed the highest overall labeling intensity in pargyline-yne hemispheres. Raw intensity values are indicated by the scale bar. (C) Representative segmented neurons in atlas space. Known pargyline-yne-bound neurons in the pons and hypothalamus were used as positive controls. Additional neuronal structures (i.e., in the medulla) were identified. The scale bar indicates raw segmented neuron density in each 10 × 10 × 10 μm voxel. (D and E) Heatmap of normalized pargyline-yne labeling intensity (D) and normalized pargyline-yne-positive neuron density (E). A total of 5 pargyline-yne-treated mice (rows) and 184 brain regions (columns) were included for analysis. For individual mice, intensity and density values were normalized by the average intensity and density of all the regions from each mouse. Purple labels indicate regions with only elevated intensity. Blue labels indicate regions with only elevated neuronal density. Red labels indicate regions with shared elevated intensity and neuronal density. Barrington’s nucleus (B*) was later found to be a false positive (see Figure S2F). The full dataset and acronyms can be found in Table S1. EPI, epithalamus; PMD, dorsal premammillary nucleus; PMV, ventral premammillary nucleus; AT, anterior tegmental nucleus; SLC, subceruleus nucleus; SLD, sublaterodorsal nucleus; AMB, nucleus ambiguus; VS, ventricular system. See also Figure S2.

The throughput and reproducibility of vCATCH across cohort-scale samples also allowed automatic and unbiased quantification of drug targets beyond visual inspection. We applied a recently developed AI-based cartography of ensembles (ACE) pipeline to analyze these datasets (Figures 2A–2C; Table S1).^52,^ After registering the 3D volumes onto the Allen Brain Atlas (ABA)^53^ (Figure S2A), we first quantified the fluorescence intensity (indicating drug binding abundance) across all brain regions (Figure 2B). Intensity quantifications revealed high drug abundance in the pons, thalamus, and midbrain (summarized in Figures 2D and S2B), for example, in the locus coeruleus (LC) and nucleus accumbens (ACB) (Figure 2D).

Leveraging the cellular-resolution imaging provided by vCATCH, we next sought to unbiasedly quantify the cell targets of pargyline. Empowered by a large training dataset and AI-based segmentation algorithms, the ACE pipeline was used to identify brain-wide pargyline-yne-positive cells (Figures 2C, S2C, and S2D). After segmentation, the cell density patterns generally agreed with fluorescence intensity profiles. Since pargyline targets both MAO-A and MAO-B with similar affinities,^43^ we performed MAO-A and MAO-B immunofluorescence and confirmed that pargyline-yne-targeted cells overlap with either or both MAO-A/B protein expression, consistent with the known affinity of pargyline for these two targets (Figure S2E).^43^

Interestingly, unbiased ACE analysis also revealed unexpected differences between fluorescence intensity and cell counts. For example, the ACB, bed nuclei of the stria terminals (BST), and the medial group of the dorsal thalamus (MED) showed extensive drug intensity, but almost no neuronal structures were detected in these areas, indicating drug binding may be associated with non-soma structures (Figures 2D, 2E, and S1E). Conversely, MAO-B-positive drug-bound cells were identified in the dorsal raphe (DR) and the anterior tegmental nucleus (AT) of the pons (Figures 2D, 2E, S1E, and S2E) despite the lack of overall fluorescence intensity increase, suggesting sparse neuronal populations were targeted by pargyline in these regions.

Taken together, by using a pargyline probe as a proof-of-concept, we demonstrated that vCATCH is compatible with clearing methods and high-throughput computational pipelines to unbiasedly quantify regional enrichment and cellular targets of covalent drugs in the mouse brain, which was not possible with traditional methods.

### vCATCH profiles whole-body targets of covalent cancer drugs

We next sought to expand vCATCH target mapping to whole animals across all organ systems. We selected two widely used cancer drugs for this study: afatinib (Gilotrif) and ibrutinib (Imbruvica), both covalent targeted kinase inhibitors (TKIs).^54,55^ Since their landmark approval in 2013, the epidermal growth factor receptor (EGFR) inhibitor afatinib and Bruton’s tyrosine kinase (BTK) inhibitor ibrutinib, respectively, have transformed the clinical landscape of non-small cell lung cancer (NSCLC)^56,57^ and B cell malignancies.^58–60^ Despite their popularity, however, there are several well-documented side effects of TKIs that are poorly understood. For example, ibrutinib is associated with cardiovascular and bleeding risks.^61,62^ Although many studies have been conducted to understand the protein targets (or off-targets) of ibrutinib,^63–67^ the *in vivo* tissue(s) or cell types responsible for such toxicities remain elusive. The ability to unbiasedly profile TKI targets *in vivo* at cellular resolution could provide crucial insight to bridge this gap.

**Figure 3.**
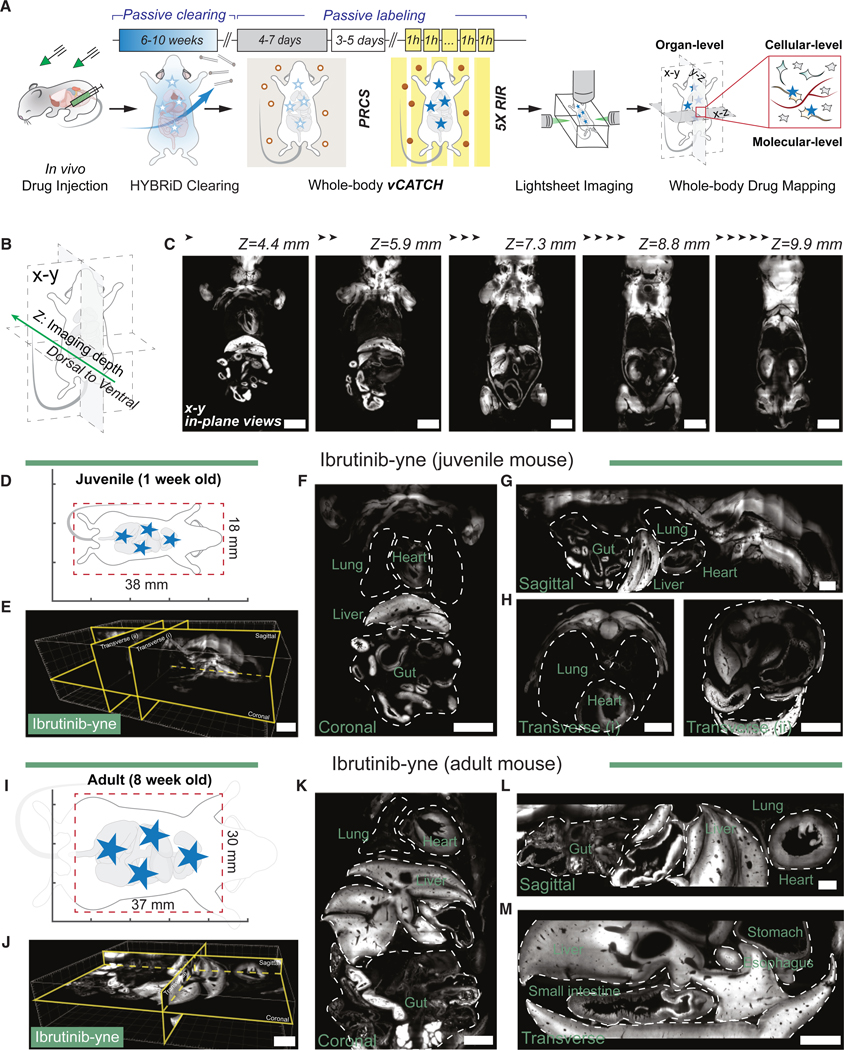
vCATCH reveals whole-body TKI distribution with high spatial resolution (A) Workflow of vCATCH whole-body labeling with estimated time of each step. (B) Schematic of imaging depth. (C) Coronal (x-y) views of vCATCH whole-body labeling in ibrutinib-yne (10 mg/kg, 1 h i.p.)-treated juvenile mouse across different z-depths. (D) Schematic of lightsheet acquisition FOV, represented by the red dotted box, for the juvenile whole body. (E) Representative ibrutinib-yne distribution overviews in a juvenile mouse with digital plane slicing in 3D volume. (F–H) Coronal (F), sagittal (G), and transverse (H) views of a juvenile mouse as shown in (E). (I) Schematic of lightsheet acquisition FOV, represented by a red dotted box, for adult mouse torso. (J) Representative ibrutinib-yne distribution overview in an adult mouse torso with digital plane slicing in a 3D volume image. (K–M) Coronal (K), sagittal (L), and transverse (M) views of adult mouse as shown in (J). Dashed lines indicate body boundary. Scale bars: 4,000 μm (C, E, F, J, and K) and 2,000 μm (G, H, L, and M). See also Figure S3.

We injected previously established clickable probes of ibrutinib-yne (10 mg/kg, i.p.) and afatinib-yne (10 mg/kg, i.p.) in wild-type mice (Figure S3A).^68^ These probes largely retain the pharmacokinetics (PK) and tissue distribution of the parent compounds (Figures S3B and S3C). We established a vCATCH protocol with 4-day PRCS and 5 rounds of RIR to homogeneously label a whole HYBRiD-cleared, 1-week-old mouse with a total volume of more than 38 × 18 × 12 mm (Figures 3A–3D). Labeling specificity was again confirmed by controlling for each vCATCH component in the lung or the spleen (canonical EGFR+ or BTK+ tissue, respectively; Figures S3D–S3G).

We then performed whole-body lightsheet imaging of these 1-week-old mouse bodies (Figures 3B–3H, S3H, and S3J–S3M) and visualized them through virtual reality (VR) (Videos S2 and S3).^69^ The performance of vCATCH in young mice motivated us to expand its capacity to adult bodies. Enhanced by 7-day PRCS and 8 rounds of RIR (Figure 3A), we labeled ibrutinib-yne in an adult mouse with a total volume of 70 × 30 × 20 mm. Although this whole volume exceeded the field of view (FOV) of most commercial light sheet systems, we were able to focus on the center of the torso (37 × 30 × 8 mm; Figure 3I) and examined drug distribution of major organs (Figures 3J–3M and S3I).

**Figure 4.**
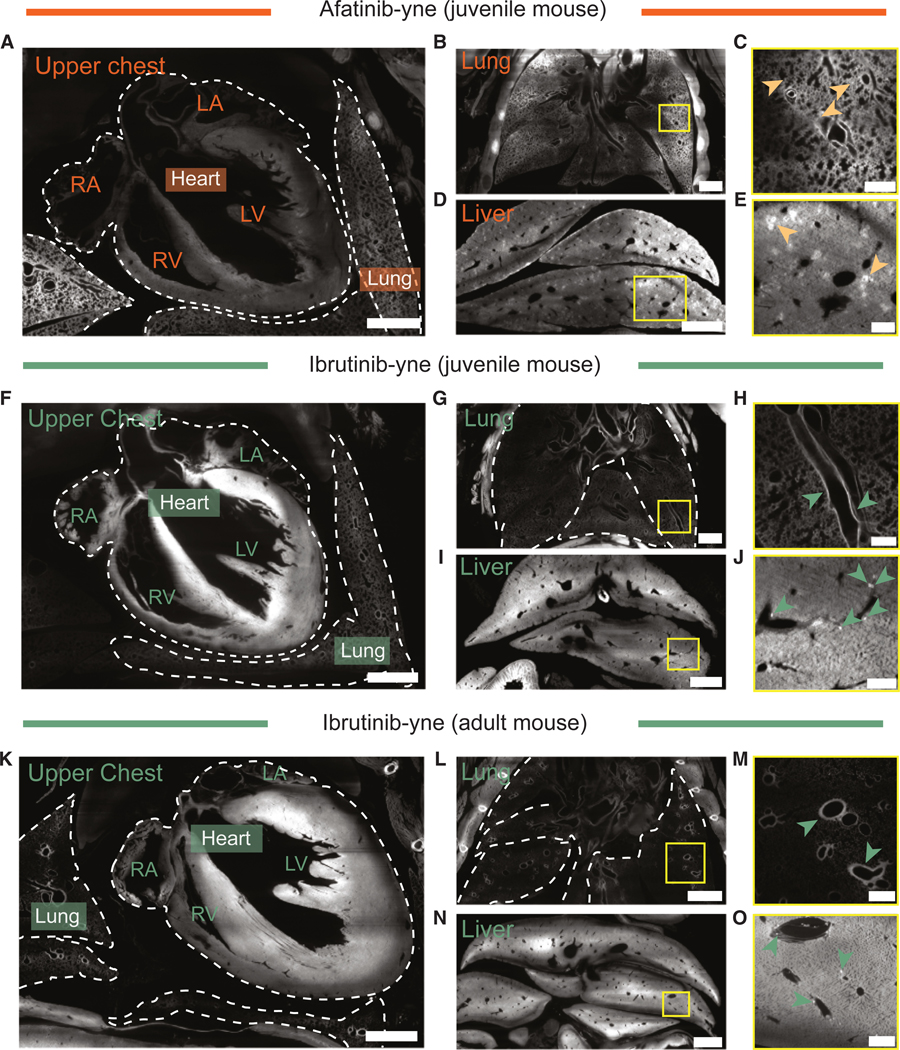
vCATCH reveals intra-organ TKI distribution (A–E) Afatinib-yne binding in the upper chest (A), lung (B and C), and liver (D and E) of juvenile mice. (F–J) Ibrutinib-yne binding in the upper chest (F), lung (G and H), and liver (I and J) of juvenile mice. (K–O) Ibrutinib-yne binding in adult mice. Figures showing the upper chest (K), lung (L and M), and liver (N and O). RA, right atrium; RV, right ventricle; LA, left atrium; LV, left ventricle. Arrows indicate drug-enriched structures. Dashed lines indicate tissue boundary. Scale bars: 1,000 μm (A, B, F, and G), 500 μm (C, D, H, I, M, and O), 100 μm (E and J), and 2,000 μm (K, L, and N). See also Figure S4.

Globally, the binding of both TKIs was highly abundant in the liver and the gastrointestinal tract (Figures 3F, 3K, S3K, and S4). In the upper chest, ibrutinib showed elevated heart binding compared with the lung, whereas the opposite enrichment was observed with afatinib (Figures 4A, 4F, and 4K). These findings are consistent with the general distributions of these two drugs based on established autoradiography data from the Food and Drug Administration (FDA).^70,71^

**Figure 5.**
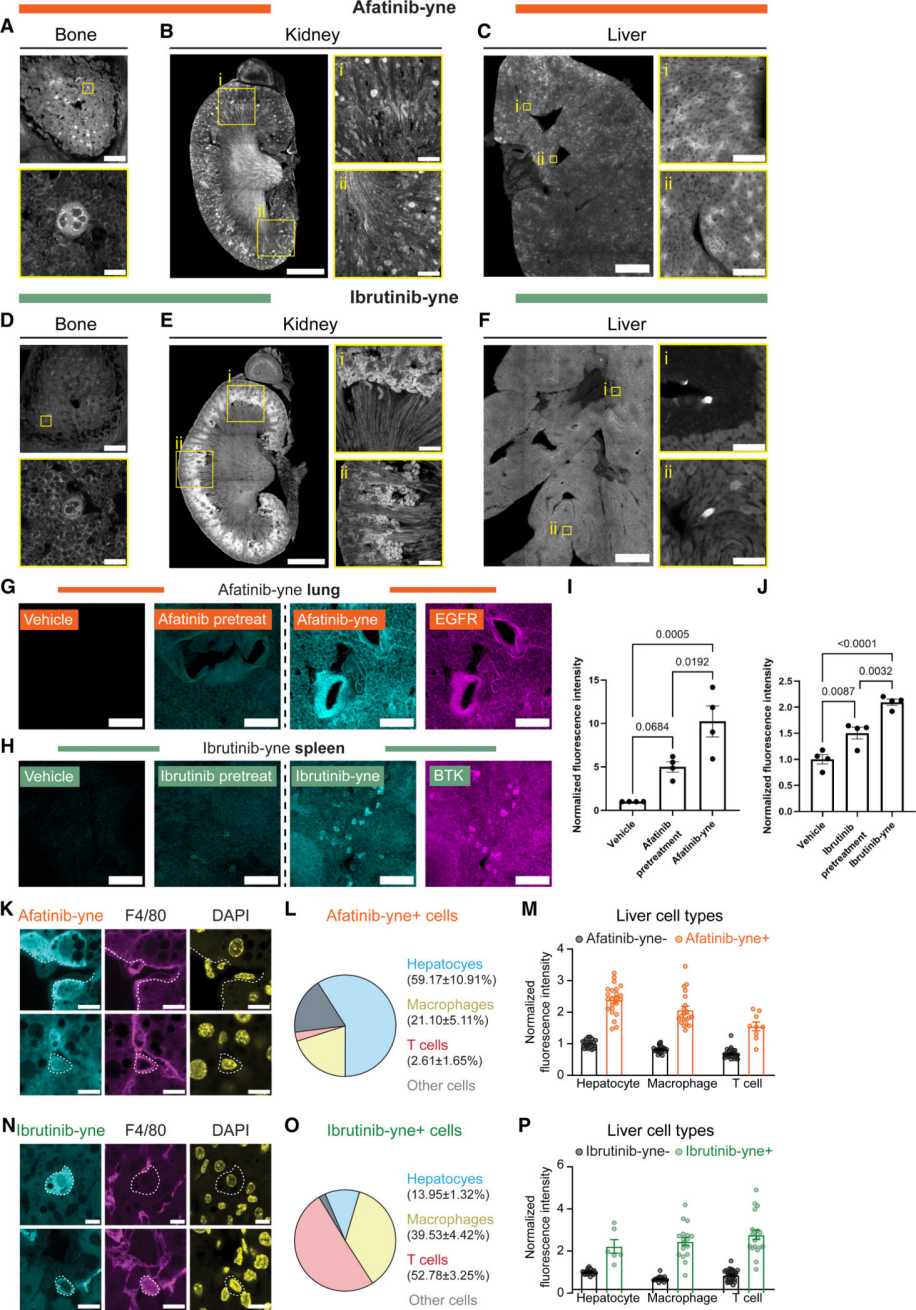
Cell-type characterization of TKI engagement in drug-enriched tissues (A–F) Afatinib-yne (A–C) and ibrutinib-yne (D–F) characterizations in the bone (A and D), kidney (B and E), and liver (C and F). (G) Afatinib pretreatment (25 mg/kg, 1 h, i.p.) in the lung co-stained with EGFR. (H) Ibrutinib pretreatment (25 mg/kg, 1 h, i.p.) in the spleen co-stained with BTK. (I and J) Quantification of afatinib-yne labeling intensity of EGFR-positive pixels in the lung (I) and ibrutinib labeling intensity of BTK-positive pixels in the spleen (J). *n* = 4 mice for each condition. One-way ANOVA with Tukey’s multiple comparisons test. *p* values plotted in the graph. (K) Representative images of afatinib-yne+ hepatocytes (top row) and afatinib-yne+ macrophages (F4/80+, bottom row). (L and M) Afatinib-yne-positive cell type (L) and cell-type-specific intensity (M) quantifications. *n =* 4 biological replicates, and 4–6 FOV were acquired for each mouse. (N) Representative images of ibrutinib-yne+ hepatocytes (top row) and ibrutinib-yne+ macrophages (F4/80+, bottom row). (O and P) Ibrutinib-yne-positive cell type (O) and cell-type-specific intensity (P) quantifications. *n =* 4 biological replicates, and 4–6 FOV were acquired for each mouse. All values are shown as mean ± SEM. Dashed lines represent the cell boundary. Detailed cell-type quantification is in Table S1. Scale bars: 200 μm (A and C, global view; B and E, zoomed-in view; G and H), 20 μm (A and C, zoomed-in view), 1,000 μm (B and E, global view), 500 μm (C and F, global view), 50 μm (C and F, zoomed-in view), and 10 μm (K and N). See also Figure S5.

The resolution of vCATCH further allowed us to examine intra-organ drug distribution in detail. In several organs, afatinib and ibrutinib displayed similar intra-organ distributions. For example, both drugs were selectively enriched in the villus in the small intestine (Figure S4). Higher-magnification confocal imaging further revealed detailed drug-bound structures across organs (Figures 5A–5F). For example, both drugs strongly bound to circular structures in the bone (Figures 5A and 5D), which were also observed in the spleen (Figure S4). Subsequent lectin staining identified these structures as vasculatures (Figures S5A and S5B). Considering that BTK expression is generally absent in blood vessels,^72^ we suspect the vasculature-associated binding may be implicated in the off-target toxicity associated with ibrutinib.^61,62^

In other organs, we observed distinct target distributions between these two drugs. For example, robust tissue enrichments in the inner stomach lining and along the nasal tract were associated with ibrutinib but not afatinib (Figures S4A and S4B). Furthermore, we discovered widely spread afatinib targets in the lung, whereas pulmonary ibrutinib labeling was restricted along the bronchi (Figures 4B, 4C, 4G, 4H, 4L, and 4M).

Interestingly, although both drugs were bound to the kidney, they displayed different intra-organ patterns (Figure S4). Afatinib was associated with glomeruli in the cortex and near the cortex-medulla interface, whereas ibrutinib was more localized to cortical tubular-like structures (Figures 5B and 5E). Similarly, our global organ-level imaging (Figures 4D, 4I, and 4N) showed that both TKIs were enriched in the liver, in agreement with the literature.^70,71^ However, higher resolution vCATCH revealed differences in their intra-organ distribution: afatinib showed crown-like signals throughout the hepatic tissue, whereas ibrutinib was associated with sparsely scattered individual cells, as demonstrated by both lightsheet (Figures 4E, 4J, and 4O) and confocal imaging (Figures 5C and 5F).

### Multiplexed molecular characterizations of drug-enriched cellular structures

Guided by the drug distribution patterns, we next sought to molecularly characterize drug-targeted cells. We first focused on afatinib binding in the lung and ibrutinib binding in the spleen based on their canonical “on-target” engagements.^72^ As expected,^68^ afatinib-yne and ibrutinib-yne signals were primarily on EGFR+ and BTK+ cells, respectively, as determined by immunostaining (Figures 5G and 5H). Importantly, pretreating the animals with the parent compound of each probe robustly reduced probe labeling (Figures 5G–5J), further confirming the specificity of these probes.

In the liver, we observed enriched yet distinct binding patterns of afatinib-yne and ibrutinib-yne (Figures 5C and 5F). We next performed molecular analysis to determine the targeted cell types of each probe. First, consistent with high EGFR expression in the hepatocytes,^72^ we found that the majority (59.17% ± 10.91%) of afatinib-bound cells were hepatocytes, followed by small fractions of macrophages (F4/80+) and T cells (CD3e+) (Figures 5K–5M and S5C). By contrast, only a small fraction (13.95% ± 1.32%) of ibrutinib-bound cells were hepatocytes, while a larger proportion (39.53% ± 4.42%) of these cells were F4/80+ macrophages (Figures 5N–5P), consistent with known BTK expression in liver macrophages.^72^ Interestingly, the largest fraction (52.78% ± 3.25%) of ibrutinib-bound cells were CD3e+ T cells despite their low BTK expression (Figures 5O and S5C–S5E),^72^ suggesting non-BTK engagement could be involved (see discussion).

Beyond the liver, we were also able to identify and quantify afatinib-enriched immune cell populations in other tissues such as the lung (Figures S5F–S5H). In summary, our vCATCH data revealed that the two TKIs are enriched in distinct cell types within individual tissues and organs, highlighting vCATCH’s capacity to profile drug targets across global, organ, tissue, and cellular scales.

## DISCUSSION

Covalent TKIs have reentered the stage for cancer treatment and broadened the horizon for the druggable space, as demonstrated by the recent developments of the mutant-selective EGFR inhibitor osimertinib and the KRAS G12C inhibitors sotorasib and adagarasib.^54^ However, despite the reignited enthusiasm, the concern for toxicity (for example, cardiotoxicity and bleeding for ibrutinib) calls for continuous innovations in characterizing their *in vivo* targets to guide future covalent drug development.

Here, by treating samples with excessive copper (PRCS) and performing multiple rounds of click reactions (RIR), we established a new method, vCATCH, which enabled robust covalent TKI mapping across the whole mouse body. This method only requires simple passive incubations and therefore offers reliability, reproducibility, and high throughput for cohort-scale biological and pharmacological analyses. Using existing pharmacokinetic data from the FDA as a reference, vCATCH confirmed organ-level drug distribution. Critically, vCATCH enabled us to identify drug engagement with intra-organ cellular resolution that is typically inaccessible through conventional methods. The capacity to resolve drug engagement across molecularly defined cell populations, tissue architectures, and organ systems, once combined with single-cell and spatial omics tools, presents transformative opportunities for spatially defined, cell-type-specific drug action characterization.

Our work so far has focused on wild-type mice for method development. By examining drug binding in healthy mice, we uncovered patterns potentially associated with off-target engagement and toxicities. For example, ibrutinib is well known to be associated with cardiac toxicity such as atrial fibrillation (AF), but the mechanism behind this association remains incompletely understood.^63–66^ Although animal studies suggested AF induction would likely require chronic administration,^65^ our study revealed clear ibrutinib enrichment in the heart after a single injection, suggesting an intrinsic enrichment of ibrutinib for the cardiovascular system. It would be of great interest to further examine ibrutinib engagement in AF models to better understand its cardiotoxicity mechanism. In addition to AF, bleeding is another major ibrutinib risk factor.^61,62^ Despite low BTK expression in endothelial cells,^72^ we observed ibrutinib engagement in the vasculature, a finding that could have implications for ibrutinib-associated hypertension and help guide subsequent molecular identification work to further characterize unique ibrutinib targets in endothelial cells.^73^

Moreover, we observed ibrutinib binding in T cells that normally do not express BTK. This binding suggested non-BTK targets of ibrutinib at the dose we used in our studies. Indeed, previous ABPP studies in Ramos cells have identified human epidermal growth factor receptor 2 (HER2)^68^ as an ibrutinib target. *In vitro* kinase activity profiling also identified interleukin-2 inducible T cell kinase (ITK) and Janus kinase 3 (JAK3) with nM affinity to ibrutinib.^67^ All of these non-BTK affinities could contribute to our observed T cell binding.

On the technology side, vCATCH represents a new strategy to use biorthogonal click chemistry to label large tissues, in contrast to most mainstream labeling methods (e.g., immunostaining, DNA/RNA hybridization, etc.) that primarily rely on biological affinities, such as antibody-antigen, biotin-streptavidin, and complementary DNA/RNA interactions. Click chemistry allows us to easily control labeling kinetics by simple buffer exchanges, achieving uniform probe penetration in ultra-large tissue without delicate procedures or apparatus. The covalent click chemistry linkage also means that the same sample can withstand harsh and prolonged processing and imaging operations, opening numerous possibilities to multiplex vCATCH with other spatial omics methods.

Finally, it is a major challenge in whole-mount tissue staining to balance the high probe concentration (such as antibody) required for tissue penetration and its associated background resulting from the nonspecific interactions between the probe and endogenous biomolecules. In this regard, the biorthogonal nature of click chemistry could better tolerate a wide range of labeling conditions without increasing noise. Currently, click chemistry-based imaging studies are primarily associated with examining transcription/translation.^74,75^ By demonstrating vCATCH’s capacity to map covalent drug binding across the entire mouse body, we propose that similar principles can be applied in ultra-large 3D tissues to visualize substrates including but not limited to protein,^76^ nucleic acid,^77^ glycan,^78^ lipid,^79^ and drug molecules.^21^ For example, a recent study (Click3D) has demonstrated that the original CATCH or similar strategies can label endogenous RNA and dyes in the mouse brain and tumor,^80^ suggesting such approaches could be adopted in future biomedical studies, although it was limited to small, individual organs.

### Limitations of the study

Focusing on method development, this study used healthy wild-type mice, in which the engagement of TKIs could differ from tumor-bearing animals. Given the robustness and simplicity of the vCATCH pipeline, however, we believe other researchers with oncology expertise could readily apply vCATCH in various cancer models. We also note that due to the size limit of the current light sheet microscopes, our imaging scope is limited to the center of adult mouse torsos. We foresee that the challenge can be addressed with emerging hardware and reconstruction pipelines.^31,81,82^ Moreover, all clickable TKI probes used in this study have been rigorously validated by published medicinal chemistry and chemoproteomics studies.^68^ However, we acknowledge that generating alkyne derivatives with similar absorption, distribution, metabolism, and excretion (ADME) properties of the original drugs requires dedicated efforts. We note that our ibrutinib-yne probe has a higher liver distribution than the parent drug, despite the latter being known to be enriched in the liver based on the FDA.^71^ This difference could confound our interpretations of ibrutinib-bound immune cells (Figure 5). Lastly, as CATCH readout can potentially reflect both on- and off-target binding and metabolite intermediates (i.e., glutathione conjugation for cysteine-targeting drugs), it is important to note that the design of future CATCH probes for other drugs must be verified by similar PK and target identification approaches, although it is becoming common to generate and characterize clickable alkyne analogs during the development of new covalent inhibitors.

## RESOURCE AVAILABILITY

### Lead contact

Further information and requests for resources and reagents should be directed to and will be fulfilled by the lead contact, Li Ye (liye@scripps.edu).

### Materials availability

All unique/stable reagents generated in this study are available from the lead contact with a completed materials transfer agreement.

### Data and code availability

All data are available in the main text or the supplementary materials. Any additional information required to reanalyze the data reported in this paper is available from the lead contact upon request. Relevant code for the study is available from https://github.com/yelabscripps/vCATCH.

## STAR★METHODS

### EXPERIMENTAL MODEL AND STUDY PARTICIPANT DETAILS

#### Mouse model

Mice were group-housed on a 12-hr light-dark cycle and fed a standard rodent chow diet. Both male and female mice were used for the study. Six to nine-week-old WT C57BL6J mice were used for vCATCH method development in 100-micron sections and hemispheres. One-week-old and eight-week-old WT C57BL6J mice were used for vCATCH in the whole mouse body. All experimental protocols were approved by the Scripps Research Institute Institutional Animal Care and Use Committee and were in accordance with the guidelines from the NIH.

### METHOD DETAILS

#### Sample collection

Alkyne drugs were administered to mice in a vehicle of 10% DMSO and 2% Tween-80 in saline for i.p. injections at the indicated dose. 1 hour (for most direct labeling experiments) or 4 hour (for pretreatment test) after injections, mice were heavily anesthetized with isoflurane and then transcardially perfused with ice-cold PBS, followed by ice-cold 4% PFA solution. Mouse organs were carefully dissected and then post-fixed in 4% PFA solution overnight at 4°C. Tissues were washed with PBS. Mouse brains were embedded in 2% agarose and sectioned with a vibratome (Leica VT1000S) at 100-micron thickness. For hemispheres, brains were cut along the midline with a razor knife. Peripheral organs from one-week-old mice were dehydrated in 30% sucrose at 4°C prior to sectioning. Samples were then carefully dried with a kimwipe and embedded in O.C.T. compound. After reaching equilibrium at −20°C, samples were cut at 100-micron thickness with a cryostat (Leica CM 1860). All obtained samples were stored in PBS with 0.02% sodium azide at 4°C for further processing.

For mice used in whole-body drug distribution labeling, the skin was carefully removed immediately after perfusion, and the peritoneum was cut down the midline to facilitate clearing and click reactions. Mice were post-fixed in 4% PFA solution for two days at °C followed by 7-day decalcification treatment in 10% EDTA/15% Imidazole at 4°C. Then, samples underwent decoloring treatment with 25% N, N, N’, N’-Tetrakis(2-Hydroxypropyl)ethylenediamine (Quadrol) in 1x PBS at 37°C with gentle agitation for 7 days. After PBS washes to remove Quadrol, mouse whole bodies were stored in PBS with 0.02% sodium azide at 4°C for further processing. For mice used in PK studies, after anesthesia, blood was collected via cardiac puncture and immediately stored in a heparin tube and placed on ice. Plasma was separated by centrifugation at 5,000 rpm for 5 min at 4°C. Next, 150 μL of a 2:1 mixture of acetonitrile/methanol was added to 50 μL of plasma. The mixture was then centrifuged at 15,000 rpm for 10 min at 4°C, and the supernatant was transferred to a liquid chromatography-mass spectrometry (LC-MS) vial. Tissue samples were dissected out and weighed. Next, tissues were homogenized in water at 250 mg/ml using a homogenizer at 4°C. The mixture was then centrifuged at 15,000 rpm for 10 min at 4°C. 50 μL of the supernatant was then collected, and 150 μL of 2:1 mixture of acetonitrile/methanol was added to the supernatant. The mixture was then centrifuged at 4°C at 15,000 rpm for 10 min, and the supernatant was transferred to an LC-MS vial. To generate a standard curve, compounds were dissolved in a 2:1:1 mixture of acetonitrile/methanol/water at 10, 1, 0.1, 0.01, 0.001 μM for MS analysis.

#### Tissue clearing

##### CLARITY

100-micron sections were cleared with CLARITY as previously described.^22,83^ Briefly, samples were incubated in A1P4 hydrogel (1% acrylamide, 0.125% Bis, 4% PFA, 0.025% VA-044 initiator (w/v), in 1X PBS at 4°C for CLARITY embedding. After overnight incubation with gentle agitation, samples were then degassed and polymerized at 37°C for 4 hours with gentle agitation. Samples were removed from hydrogel and washed with 6% PBS-SDS (pH=7.0) at 37°C for two days. After clearing, samples were washed with PBST (pH=7.0 with 0.2% Triton-X100, same for the following) three times (10 min each time) at RT to remove residue SDS. Samples were briefly washed with PBS and then stored in PBS with 0.02% sodium azide at 4°C.

##### HYBRiD

Mouse hemispheres and bodies were cleared with HYBRiD as previously described.^27^ In brief, samples were dehydrated in an increasing THF/25% Quadrol gradient (50%, 70%, 80%, 100%, 100%; 1 hour per step for hemispheres, 3–12 hours per step for whole bodies), delipidated three times in DCM (1 hour each for hemispheres, 3–12 hours each for whole bodies), and rehydrated in a decreasing THF/25% Quadrol gradient (100%, 100%, 80%, 70%, 50%; 0.5 hour per step for hemispheres, 1.5–6 hours per step for whole bodies). After clearing, samples were extensively washed with PBS to remove residual organic solvent before hydrogel embedding. Samples were equilibrated in A1P4 hydrogel (3 days for hemispheres, 7–10 days with one refreshment for whole bodies) at 4°C on a shaker, then degassed and polymerized at 37°C for 4 hours with gentle agitation. Thereafter, samples were passively cleared with LiOH Boric Buffer with 6% SDS (pH=9) until transparent (1 week for hemispheres, 6–10 weeks for whole bodies). Samples were extensively washed in PBST to remove residual SDS and then stored in PBS with 0.02% sodium azide at 4°C.

#### Click labeling in tissues

##### CATCH in 100-micron sections

100-micron sections were labeled with an updated CATCH protocol. Unless otherwise noted, sections were incubated in 10 mM CuSO_4_ in H_2_O at RT overnight. Sections were then transferred to CATCH reaction buffer containing 5 μM AZDye 647-picolyl azide, 150 μM CuSO4, 300 μM BTTP, 2.5 mM NaAsb, and 10% DMSO in 1X PBS. After 1 hour of reaction in the dark with minor agitation, samples were washed with buffer containing 4 mM EDTA in PBST (PBST-EDTA, same for the following) at RT for 3×10 min. Tissue samples were then stained with DAPI (1:3000 dilution in PBST from 10 mM stock) for 15 mins at RT. Samples were then either used for refractive index (RI) matching by RapiClear and confocal imaging or proceeded to further immunolabeling for cell-type registration.

##### vCATCH in hemispheres

For hemisphere labeling, each hemisphere was first pre-incubated with 25 mL of 10 mM CuSO4 in H_2_O for one day at RT for PRCS. Hemispheres were transferred to vCATCH incubation buffer (5 μM AZDye 647-picolyl azide, 1 mM CuSO4, 2 mM BTTP, 10% DMSO in 1X PBS, 3 mL for each hemisphere) for 1 day at RT. Hemispheres were then transferred to the newly prepared full vCATCH reaction buffer (5 μM AZDye 647-picolyl azide, 1 mM CuSO4, 2 mM BTTP, 25 mM NaAsb, 10% DMSO in 1X PBS, 3 mL for each hemisphere) for click labeling. The vCATCH reaction buffer was refreshed every hour for two rounds of RIR. Hemispheres were washed with PBST-EDTA at RT. The washing buffer was refreshed at the end of the day and then daily until it became colorless. The hemispheres were then washed with PBST for 1 hour at RT. After washing, hemispheres were cut into 100-micron coronal sections for labeling depth characterizations. Alternatively, hemispheres were carefully dried with a Kimwipe and then incubated in 3 mL EasyIndex for RI matching and lightsheet imaging.

##### vCATCH in the whole mouse

Cleared mouse bodies were placed in 500 mL per mouse of 10 mM CuSO_4_ in H_2_O with agitation for 4–7 days at RT with daily buffer refreshes. After PRCS, whole-body samples were individually incubated in 10–40 mL of vCATCH incubation buffer for 3–5 days on a shaker at RT with daily buffer changes. Samples were then transferred to vCATCH reaction buffer (10–40 mL each) to initiate the reaction. Five to eight successive rounds of 1-hour click reactions were performed to thoroughly label the entire tissue depth. After click labeling, samples were washed with PBST-EDTA with daily refreshments until the buffer was colorless. After an additional day of PBST wash, mouse bodies were carefully dried with a Kimwipe and then thoroughly immersed in 25–40 mL EasyIndex at 37°C for thorough RI matching in preparation for lightsheet imaging.

#### Immunostaining

CATCH-labeled 100-micron sections were incubated with primary antibodies 1:200 diluted in PBST overnight at 4°C. Samples were then washed with PBST 3 × 30 min at RT prior to incubation with secondary antibodies diluted 1:600 in PBST. Samples were incubated with secondary antibodies overnight and then washed with PBST 3 × 30 min at RT.

#### Confocal microscopy

100-micron samples were mounted with RapiClear for RI matching and imaged with an Olympus FV3000 confocal microscope. To characterize CATCH labeling depth in 100-micron sections, samples were imaged at the primary somatosensory cortex layer V with a 10X, 0.6 NA, water immersion objective (XLUMPlanFI, Olympus) at 0.414 micron/pixel resolution and a step size of 10 microns. To examine hemisphere labeling depth and global drug distribution within individual organs, samples were imaged with a 10X, 0.6 NA, water immersion objective (XLUMPlanFI, Olympus) at 2.49 microns/pixel resolution and a step size of 10 microns. For cell type characterizations, samples were imaged at the top surface with a 40X, 1.25 NA, silicone oil objective (UPlanSApo, Olympus) at 0.094 micron/pixel resolution and a step size of 3 microns. All confocal data were saved in TIFF format for further analysis.

#### Lightsheet microscopy

Whole hemisphere/body samples were imaged using a lightsheet microscope from LifeCanvas Technologies. RI-matched whole hemisphere and body samples were mounted in 1% agarose/Easy Index for imaging. Lightsheet imaging was performed in a SmartSPIM chamber with Easy Index matched immersion oil (RI=1.52). A 3.6x objective was used with 0.28 NA, 1.8μm/1.8μm/2μm *xyz* voxel size. Imaging was performed by illuminating samples along the bilateral plane of symmetry. Lightsheet raw data was then destriped, stitched, and saved as TIFF format for further conversion.

#### Drug PK analysis

Targeted measurements were performed using an Agilent 6470 triple quadrupole LC-MS instrument. MS analysis was performed using AJS in positive mode. The AJS source parameters were set as follows: the dry gas temperature was set at 250 °C with a gas flow of 12 l/min and the nebulizer pressure at 25 psi; the sheath gas temperature was set to 300 °C with the sheath gas flow set at 12 l/min; and the capillary voltage was set to 3,500 V. Separation of compound was conducted using a ZORBAX RR Eclipse Plus C18 95Å LC column (Agilent 959961–902) with reversed-phase chromatography and the column temperature was maintained at 30 °C. Mobile phases were as follows: buffer A, 100% water with 0.1% formic acid; buffer B, 100% acetonitrile with 0.1% formic acid. The flow rate for each run started at 95% A for 9 minutes at 0.7 ml/min, followed by a gradient starting at 95% A, changing linearly to 5% A / 95% B over the course of 12 minutes at 0.7 ml/min. The flow rate was maintained at 5% A / 95% for 6 minutes at 0.7 ml/min. The last 3 minutes consisted of a re-equilibration back to 95% A / 5% B at 0.7 ml/min. Multiple reaction monitoring was performed for the indicated chemicals with the listed dwell times, fragmentor voltage, collision energies, cell accelerator voltages and polarity. We selected previously reported transitions for the quantification of ibrutinib^84^ and afatinib.^85^ The MS ionization parameters for the targeted metabolomics are presented in Table S1. Quantification of the compound concentrations was performed by generating an external standard curve with known concentrations of each compound. Compound standards were analyzed alongside the biological samples using the same targeted triple quadrupole LC/MS method in the same run. A calibration standard curve generated from the compound standard concentrations and total peak areas were used to calculate the concentrations of each compound. It is worth noting that, contrary to the high liver distribution from the autoradiography study,^71^ our PK data showed overall lesser ibrutinib liver enrichment (Figure S3C). The caveat may be due to the different detection analytes (isotope tag vs. intact free drug). And the observed ibrutinib liver enrichment may reflect strong local metabolism and potential off-target binding.

### QUANTIFICATION AND STATISTICAL ANALYSIS

#### Hemisphere labeling depth characterization

Hemispheres were sectioned coronally as 100 micron sections and imaged to characterize vCATCH labeling depth. Images were first processed as maximum intensity projections. A rectangular area (805 × 50 pixels, 2001.17 × 136.73 microns) was drawn from the cortical outer layer inwards (from tissue surface to tissue center) as indicated in Figure 1A. The signal profile was plotted using the ‘Plot Profile’ function in ImageJ as reported.^27,86,87^ The fluorescence signal profile was then normalized by the intensity value at the start of the line.

#### Thin section labeling depth characterization

CATCH labeling intensity across the 100-micron section was quantified as previously reported.^21,27,86^ Briefly, a 150 × 150 pixel (62.1 × 62.1 micron) ROI surrounding a labeled neuron was generated at z = 10 μm and 50 μm at cortex layer V, and an auto threshold was applied to measure the mean drug-positive pixel intensity as *I*_signal_. The mean intensity of the remaining pixels was used *I*_background_. The mean labeling intensity was defined as *I*_labeling_ = *I*_signal_-*I*_background_.

#### Cell type-specific characterization

CD3e, F4/80, CD4, CD8, and CD19 positive cells were characterized by immune staining. Hepatocytes were identified based on their large size (20–30 microns), round/oval-shaped nucleus, and hexagonal morphology by well-trained experts. To minimize bias, individual fields of view (FOV) were first acquired based on the DAPI and the immunostaining channel without knowledge of the CATCH/drug channel (with the exception of CD3e+ibrutinib+ cells, where the CD4 and CD8 channel were kept blind during imaging acquisition) to assign cell types. The CATCH positivity was post-hoc assigned to individual cells after their cell types have been determined, followed by overlapping quantifications. For intensity characterizations, individual cells were selected along the boundary and cropped out for quantification. A threshold was applied in the drug channel to analyze CATCH labeling intensity in individual cells of interest. The full dataset of each cell population can be found in Table S1.

#### Global drug intensity characterization

To measure global drug intensity across intact thin tissue sections, confocal images were first processed as maximum intensity projections. A threshold was then applied in the counterstain channel (DAPI or target immunostaining) to cover the whole tissue or target positive pixels. The threshold area was selected as tissue containing pixels. The average drug channel intensity in the selected pixels was then quantified as global drug intensity.

#### Volumetric drug binding visualization

Lightsheet imaged hemispheres and whole body images were converted into Imaris files and visualized via Imaris 10.2.0. The hemisphere drug labeling video was generated by Imaris. For VR-based whole-body drug visualization, data was converted by syGlass, and videos were made with a Meta Quest 3 glass. For hemisphere coronal and transverse views, TIFF files were imported as image sequences and then resliced by Fiji ImageJ. For drug distribution visualization across organs, TIFF files were imported as image sequences and sampled at 100-micron step size. Individual organ of interest was then cropped out for visualization.

#### Automated Brain-wide Cell Mapping & Signal Intensity Calculation

We employed an AI-based Cartography of Ensembles (ACE) pipeline to generate whole-brain segmentation maps of pargylineyne+ cells. ACE is an open-source 3D deep learning pipeline (available through the MIRACL platform^88^; https://miracl.readthedocs.io/) to map local cell activations using tera-voxel size light sheet microscopy datasets. Given the significant differences in morphological features between pargyline-yne+ cells and cFos+ cells (which the ACE models were originally trained on), we first fine-tuned the ACE vision transformer (ViT) model. For fine-tuning, we randomly selected 225 image patches of 96^3^ voxels each (0.17×0.17×0.19 mm^3^) from two subjects in the dataset and generated ground truth using the Labkit annotator^89^ in ImageJ/Fiji software.^90^ We fine-tuned the ViT model on half of these patches and used the other half for evaluation. The model was fine-tuned for 200 epochs using the Adam optimizer^91^ with an initial learning rate of 0.0001 and an equally weighted Dice-Cross Entropy loss function. To account for artifacts in the dataset, the cross-entropy calculation for “background” voxels was weighted 2× higher than for voxels annotated as pargyline-yne+ cells. The best model, which achieved an average Dice score of 0.7 on the validation dataset, was used to generate segmentation maps for the entire dataset. Subsequently, a shape filter was applied to the segmentation maps to eliminate false positives such as vasculature traces in cortical regions using the shape filter plugin in ImageJ/Fiji.^92^ To distinguish individual cells, a connected component analysis was applied to the 3D segmentation maps of each subject. Following segmentation, we automatically registered the light sheet hemisphere data to the ABA using the MIRACL platform.^53,88^ The accuracy of the registration and warping were evaluated using quality control checkpoints. Deformation fields obtained from the registration algorithm were used to warp the ABA labels to the native space of each subject. Finally, we computed the number of cells within each region at depth 6 (labels grouped to a maximum atlas ontology depth of 6, by combining finer labels under their parent labels), by identifying the coordinates of the center of each cell.

To map significant localized group-wise differences in pargyline-yne+ cells in an atlas-agnostic manner, we employed ACE’s cluster-wise threshold-free cluster enhancement permutation test using a group-wise ANOVA. The segmentation maps obtained by the deep learning models were downsampled to ABA 10-μm resolution while minimizing information loss by applying a convolution-based voxelization procedure to the whole-brain segmentation maps (based on our prior work^88^). Following voxelization, segmentation maps were aligned with the ABA at a 10-μm resolution using deformation fields obtained via the registration module. The voxelated and warped segmentation maps were then passed to the ACE statistical module (using default parameters), which produced a cluster-wise p-value map representing significant (alpha < 0.05 after correction) localized cell activation between the pargyline-yne treated and control groups.

We computed signal intensity information for each ABA label using the “*miracl lbls stats*” function in our MIRACL platform, which processes NIfTI images and their registered warped ABA labels for each subject. This function extracts key intensity metrics, such as the average, minimum, and maximum voxel intensity, across the input volumes for each label. We further summarized it by grouping labels at depth 6 using the atlas ontology.

#### Brain-wide drug binding heatmap visualization

Brain regions with positive cell counts in all 5 pargyline-yne-treated hemispheres were included for heatmap generation. Cell density (number of cells per mm^3^) was obtained by dividing cell count values by the volume of the brain region. Intensity and cell density were then normalized by the average values in individual hemispheres. Heatmaps were generated using the Python function ‘sns.heatmap’^93^, with each region displaying the normalized intensity and density across different subjects. The height of each subplot, corresponding to different regions, is proportional to the number of areas within that region. Code for generating heatmaps is available via https://github.com/yelabscripps/vCATCH.

All statistical analysis was performed with Prism X10. Detailed tests used and results are indicated in figure legends and available in Table S1.

## Supplementary Material

Table S1

Video S1

Video S2

Video S3

1

Supplemental information can be found online at https://doi.org/10.1016/j.cell.2025.11.030.

## Figures and Tables

**Table:** KEY RESOURCES TABLE

REAGENT or RESOURCE	SOURCE	IDENTIFIER

Antibodies		

anti-CD19	Novus Biologicals	Cat#NBP2–25196; RRID: AB_3274353
anti-CD3e	abcam	Cat#ab16669; RRID: AB_443425
anti-F4/80	Thermo Scientific	Cat#14–4801-82; RRID: AB_467558
anti-CD4	Thermo Scientific	Cat#14–9766-82; RRID: AB_2573008
anti-CD8	Biolegend	Cat#100702; RRID: AB_312741
anti-MAO-A	Proteintech	Cat#10539–1-AP; RRID: AB_2137251
anti-MAO-B	Thermo Scientific	Cat#PA5–28338; RRID: AB_2545814
anti-EGFR	abcam	Cat#ab52894; RRID: AB_869579
anti-BTK	Thermo Scientific	Cat#PA5–20085; RRID: AB_11153734
DyLight 488 lectin	Vector Laboratories	Cat#DL-1174; RRID: AB_2336404
Alexa Fluor 488 F(ab’)2 Fragment Donkey anti-Mouse IgG	Jackson Immuno Research	Cat#715–546-150; RRID: AB_2340849
Alexa Fluor 594 F(ab’)2 Fragment Donkey anti-Rat IgG	Jackson Immuno Research	Cat#712–585-153; RRID: AB_2340689
Alexa Fluor 594 F(ab’)2 Fragment Donkey anti-Mouse IgG	Jackson Immuno Research	Cat#715–586-150; RRID: AB_2340857
Alexa Fluor 488 F(ab’)2 Fragment Donkey anti-Rabbit IgG	Jackson Immuno Research	Cat#711–546-152; RRID: AB_2340619
Alexa Fluor 488 F(ab’)2 Fragment Donkey anti-Rat IgG	Jackson Immuno Research	Cat#712–546-153; RRID: AB_2340686

Chemicals, peptides, and recombinant proteins		

AZDye 647 Picolyl Azide	Vector laboratories	Cat#CCT-1300
3-[4-({bis[(1-tert-butyl-1H-1,2,3-triazol-4-yl)methyl]amino}methyl)-1H-1,2,3-triazol-1-yl] propanol (BTTP)	Vector laboratories	Cat# CCT-1414; CAS**#**1334179–85-9
DMSO	Sigma-Aldrich	Cat#D8418; CAS#67–68-5
Copper sulfate	Sigma Aldrich	Cat#C1297; CAS#7758–98-7
Sodium ascorbate	Sigma Aldrich	Cat#A4034; CAS#134–03-2
PF7845-yne	Niphakis et al.^44^	N/A
Pargyline-yne	Krysiak et al.^43^	N/A
Afatinib-yne (PF-06672131)	Sigma Aldrich	Cat#PZ0243, CAS# 1621002–27-4
Ibrutinib-yne (PF-06658607)	Sigma Aldrich	Cat#PZ0242, CAS# 1621002–24-1
DAPI	Sigma-Aldrich	Cat#D9542; CAS#28718–90-3
RapiClear, RI=1.45	Sunjin Lab	Cat#RCCS005
EasyIndex, RI=1.52	LifeCanvas Technologies	Cat#EI-500–1.52
EasyIndex Matched Immersion Oil, RI=1.52	LifeCanvas Technologies	Cat#Oil-1.52
Phosphate Buffered Saline (PBS), 10X, pH 7.2	Fisher Scientific	Cat#70–013-032
Ethylenediaminetetraacetic acid (EDTA), BioUltra, anhydrous, >=99% (titration)	Sigma-Aldrich	Cat#EDS; CAS#60–00-4
EDTA (0.5 M), pH 8.0, RNase-free	Thermo Scientific	Cat#AM9261
Triton^™^ X-100,BioXtra	Sigma-Aldrich	Cat#T9284–500ML; CAS#9036–19-5
40% acrylamide solution	Bio-Rad	Cat#1610140
2% Bis Solution	Bio-Rad	Cat#1610142
EMS 32% Paraformaldehyde (PFA) Aqueous Solution	Electron Microscopy Sciences	Cat#15714-S
VA-044	Fisher Scientific	Cat#NC0632395; CAS#27776–21-2
4% PFA solution	Fisher Scientific	Cat#5033441
Tetrahydrofuran (THF)	Sigma-Aldrich	Cat#186562; CAS# 109–99-9
Dichloromethane (DCM)	Sigma-Aldrich	Cat#270997; CAS# 75–09-2
Acetonitrile, Optima^™^ LC/MS Grade	Fisher Scientific	Cat#A9554; CAS#75–05-8
Methanol, Optima^™^ LC/MS Grade	Fisher Scientific	Cat#A4564; CAS#67–56-1
Water, Optima^™^ LC/MS Grade	Fisher Scientific	Cat#W64; CAS#7732–18-5
ReagentPlus^®^ Imidazole, Crystalline, 99%	Sigma Aldrich	Cat#I202–500G; CAS**#**288–32-4
N,N,N’,N’-Tetrakis(2-Hydroxypropyl)ethylenediamine (Quadrol), Liquid, 98%	Sigma Aldrich	Cat#122262–1L; CAS#102–60-3
Sodium dodecyl sulfate (SDS) 20% solution	Research Products International	Cat# L23100
Lithium hydroxide monohydrate	Sigma Aldrich	Cat#402974; CAS#1310–66-3
Boric acid	Sigma Aldrich	Cat#B6768–1KG; CAS#10043–35-3
Sodium azide	Sigma Aldrich	Cat#S2002–100G; CAS#26628–22-8
Agarose	Sigma Aldrich	Cat#A6013–500G; CAS#9012–36-6
Sucrose	Sigma Aldrich	Cat#S8501–5KG; CAS#57–50-1
Tissue-Tek^®^ O.C.T. Compound	Sakura Finetek USA Inc	Cat#62550–12

Deposited data		

Brain wide ACE analysis	This paper	https://github.com/yelabscripps/vCATCH

Experimental models: Organisms/strains		

Mouse: C57BL6J	Jackson Laboratory	#000664

Software and algorithms		

Fiji-ImageJ	Fiji	https://imagej.net/software/fiji/
PrismX10	Graphpad	https://www.graphpad.com/
Imaris10.2.0	OXFORD INSTRUMENTS	https://imaris.oxinst.com/
syGlass v1.8.2	syGlass	https://www.syglass.io/
FreeSurfer 8.0.0	FreeSurfer	https://surfer.nmr.mgh.harvard.edu/fswiki/DownloadAndInstall
Miracle	Miracle	https://miracl.readthedocs.io/en/latest/

## Highlights

- Copper saturation and repeated reactions enabled 3D whole-body click labeling
- vCATCH enabled unbiased and registered drug-binding profiling in the mouse brain
- vCATCH revealed cellular binding of covalent cancer drugs across the adult mouse body

## References

[1] Huang Z, Ogasawara D, Seneviratne UI, Cognetta AB 3rd, Am Ende CW, Nason DM, Lapham K, Litchfield J, Johnson DS, and Cravatt BF (2019). Global Portrait of Protein Targets of Metabolites of the Neurotoxic Compound BIA 10–2474. ACS Chem. Biol. 14, 192–197. 10.1021/acschembio.8b01097. 30702848 PMC6383364

[2] Chang JW, Cognetta AB 3rd, Niphakis MJ, and Cravatt BF (2013). Proteome-wide reactivity profiling identifies diverse carbamate chemotypes tuned for serine hydrolase inhibition. ACS Chem. Biol. 8, 1590–1599. 10.1021/cb400261h. 23701408 PMC3806897

[3] Van Esbroeck ACM, Janssen APA, Cognetta AB, Ogasawara D, Shpak G, Van Der Kroeg M, Kantae V, Baggelaar MP, De Vrij FMS, Deng H, (2017). Activity-based protein profiling reveals off-target proteins of the FAAH inhibitor BIA 10–2474. Science 356, 1084–1087. 10.1126/science.aaf7497. 28596366 PMC5641481

[4] Reinecke M, Brear P, Vornholz L, Berger BT, Seefried F, Wilhelm S, Samaras P, Gyenis L, Litchfield DW, Médard G, (2024). Chemical proteomics reveals the target landscape of 1,000 kinase inhibitors. Nat. Chem. Biol. 20, 577–585. 10.1038/s41589-023-01459-3. 37904048 PMC11062922

[5] Offensperger F, Tin G, Duran-Frigola M, Hahn E, Dobner S, Ende CWA, Strohbach JW, Rukavina A, Brennsteiner V, Ogilvie K, (2024). Large-scale chemoproteomics expedites ligand discovery and predicts ligand behavior in cells. Science 384, eadk5864. 10.1126/science.adk5864. 38662832

[6] Shalem O, Sanjana NE, Hartenian E, Shi X, Scott DA, Mikkelson T, Heckl D, Ebert BL, Root DE, Doench JG, (2014). Genome-scale CRISPR-Cas9 knockout screening in human cells. Science 343, 84–87. 10.1126/science.1247005. 24336571 PMC4089965

[7] Zhao J, Tang Z, Selvaraju M, Johnson KA, Douglas JT, Gao PF, Petrassi HM, Wang MZ, and Wang J (2022). Cellular Target Deconvolution of Small Molecules Using a Selection-Based Genetic Screening Platform. ACS Cent. Sci. 8, 1424–1434. 10.1021/acscentsci.2c00609. 36313155 PMC9615120

[8] Li H, Ma T, Remsberg JR, Won SJ, DeMeester KE, Njomen E, Ogasawara D, Zhao KT, Huang TP, Lu B, (2023). Assigning functionality to cysteines by base editing of cancer dependency genes. Nat. Chem. Biol. 19, 1320–1330. 10.1038/s41589-023-01428-w. 37783940 PMC10698195

[9] Shi J, Wang E, Milazzo JP, Wang Z, Kinney JB, and Vakoc CR (2015). Discovery of cancer drug targets by CRISPR-Cas9 screening of protein domains. Nat. Biotechnol. 33, 661–667. 10.1038/nbt.3235. 25961408 PMC4529991

[10] Perrin J, Werner T, Kurzawa N, Rutkowska A, Childs DD, Kalxdorf M, Poeckel D, Stonehouse E, Strohmer K, Heller B, (2020). Identifying drug targets in tissues and whole blood with thermal-shift profiling. Nat. Biotechnol. 38, 303–308. 10.1038/s41587-019-0388-4. 31959954

[11] Liu Z, Wang P, Wold EA, Song Q, Zhao C, Wang C, and Zhou J (2021). Small-Molecule Inhibitors Targeting the Canonical WNT Signaling Pathway for the Treatment of Cancer. J. Med. Chem. 64, 4257–4288. 10.1021/acs.jmedchem.0c01799. 33822624

[12] Ji H, Lu X, Zhao S, Wang Q, Liao B, Bauer LG, Huber KVM, Luo R, Tian R, and Tan CSH (2023). Target deconvolution with matrix-augmented pooling strategy reveals cell-specific drug-protein interactions. Cell Chem. Biol. 30, 1478–1487.e7. 10.1016/j.chembiol.2023.08.002. PMC10840709 37652024

[13] Savitski MM, Reinhard FBM, Franken H, Werner T, Savitski MF, Eberhard D, Martinez Molina D, Jafari R, Dovega RB, Klaeger S, (2014). Tracking cancer drugs in living cells by thermal profiling of the proteome. Science 346, 1255784. 10.1126/science.1255784. 25278616

[14] Stickels RR, Murray E, Kumar P, Li J, Marshall JL, Di Bella DJ, Arlotta P, Macosko EZ, and Chen F (2020). Highly sensitive spatial transcriptomics at near-cellular resolution with Slide-seqV2. Nat. Biotechnol. 39, 313–319. 10.1038/s41587-020-0739-1. 33288904 PMC8606189

[15] Rodriques SG, Stickels RR, Goeva A, Martin CA, Murray E, Vanderburg CR, Welch J, Chen LM, Chen F, and Macosko EZ (2019). Slide-seq: A scalable technology for measuring genome-wide expression at high spatial resolution. Science 363, 1463–1467. 10.1126/science.aaw1219. 30923225 PMC6927209

[16] Bhatia HS, Brunner A-D, Öztürk F, Kapoor S, Rong Z, Mai H, Thielert M, Ali M, Al-Maskari R, Paetzold JC, (2022). Spatial proteomics in three-dimensional intact specimens. Cell 185, 5040–5058.e19. 10.1016/j.cell.2022.11.021. 36563667

[17] Unterauer EM, Shetab Boushehri S, Jevdokimenko K, Masullo LA, Ganji M, Sograte-Idrissi S, Kowalewski R, Strauss S, Reinhardt SCM, Perovic A, (2024). Spatial proteomics in neurons at single-protein resolution. Cell 187, 1785–1800.e16. 10.1016/j.cell.2024.02.045. 38552614

[18] Hickey JW, Neumann EK, Radtke AJ, Camarillo JM, Beuschel RT, Albanese A, McDonough E, Hatler J, Wiblin AE, Fisher J, (2021). Spatial mapping of protein composition and tissue organization: a primer for multiplexed antibody-based imaging. Nat. Methods 19, 284–295. 10.1038/s41592-021-01316-y. 34811556 PMC9264278

[19] Shi H, He Y, Zhou Y, Huang J, Maher K, Wang B, Tang Z, Luo S, Tan P, Wu M, (2023). Spatial atlas of the mouse central nervous system at molecular resolution. Nature 622, 552–561. 10.1038/s41586-023-06569-5. 37758947 PMC10709140

[20] Zeng H, Huang J, Ren J, Wang CK, Tang Z, Zhou H, Zhou Y, Shi H, Aditham A, Sui X, (2023). Spatially resolved single-cell translatomics at molecular resolution. Science 380, eadd3067. 10.1126/science.add3067. PMC11146668 37384709

[21] Pang Z, Schafroth MA, Ogasawara D, Wang Y, Nudell V, Lal NK, Yang D, Wang K, Herbst DM, Ha J, (2022). In situ identification of cellular drug targets in mammalian tissue. Cell 185, 1793–1805.e17. 10.1016/j.cell.2022.03.040. 35483372 PMC9106931

[22] Sylwestrak EL, Rajasethupathy P, Wright MA, Jaffe A, and Deisseroth K (2016). Multiplexed Intact-Tissue Transcriptional Analysis at Cellular Resolution. Cell 164, 792–804. 10.1016/j.cell.2016.01.038. 26871636 PMC4775740

[23] Murray E, Cho JH, Goodwin D, Ku T, Swaney J, Kim SY, Choi H, Park YG, Park JY, Hubbert A, (2015). Simple, Scalable Proteomic Imaging for High-Dimensional Profiling of Intact Systems. Cell 163, 1500–1514. 10.1016/j.cell.2015.11.025. 26638076 PMC5275966

[24] Mai H, Luo J, Hoeher L, Al-Maskari R, Horvath I, Chen Y, Kofler F, Piraud M, Paetzold JC, Modamio J, (2023). Whole-body cellular mapping in mouse using standard IgG antibodies. Nat. Biotechnol. 42, 617–627. 10.1038/s41587-023-01846-0. 37430076 PMC11021200

[25] Wang X, Allen WE, Wright MA, Sylwestrak EL, Samusik N, Vesuna S, Evans K, Liu C, Ramakrishnan C, Liu J, (2018). Three-dimensional intact-tissue sequencing of single-cell transcriptional states. Science 361, eaat5691. 10.1126/science.aat5691. PMC6339868 29930089

[26] Tyler DS, Vappiani J, Cañeque T, Lam EYN, Ward A, Gilan O, Chan Y-C, Hienzsch A, Rutkowska A, Werner T, (2017). Click chemistry enables preclinical evaluation of targeted epigenetic therapies. Science 356, 1397–1401. 10.1126/science.aal2066. 28619718 PMC5865750

[27] Nudell V, Wang Y, Pang Z, Lal NK, Huang M, Shaabani N, Kanim W, Teijaro J, Maximov A, and Ye L (2022). HYBRiD: hydrogel-reinforced DISCO for clearing mammalian bodies. Nat. Methods 19, 479–485. 10.1038/s41592-022-01427-0. 35347322 PMC9337799

[28] Murakami TC, Mano T, Saikawa S, Horiguchi SA, Shigeta D, Baba K, Sekiya H, Shimizu Y, Tanaka KF, Kiyonari H, (2018). A three-dimensional single-cell-resolution whole-brain atlas using CUBIC-X expansion microscopy and tissue clearing. Nat. Neurosci. 21, 625–637. 10.1038/s41593-018-0109-1. 29507408

[29] Kim SY, Cho JH, Murray E, Bakh N, Choi H, Ohn K, Ruelas L, Hubbert A, McCue M, Vassallo SL, (2015). Stochastic electrotransport selectively enhances the transport of highly electromobile molecules. Proc. Natl. Acad. Sci. USA 112, E6274–E6283. 10.1073/pnas.1510133112. 26578787 PMC4655572

[30] Tainaka K, Murakami TC, Susaki EA, Shimizu C, Saito R, Takahashi K, Hayashi-Takagi A, Sekiya H, Arima Y, Nojima S, (2018). Chemical Landscape for Tissue Clearing Based on Hydrophilic Reagents. Cell Rep. 24, 2196–2210.e9. 10.1016/j.celrep.2018.07.056. 30134179

[31] Park J, Wang J, Guan W, Gjesteby LA, Pollack D, Kamentsky L, Evans NB, Stirman J, Gu X, Zhao C, (2024). Integrated platform for multiscale molecular imaging and phenotyping of the human brain. Science 384, eadh9979. 10.1126/science.adh9979. PMC11830150 38870291

[32] Ku T, Guan W, Evans NB, Sohn CH, Albanese A, Kim J-G, Frosch MP, and Chung K (2020). Elasticizing tissues for reversible shape transformation and accelerated molecular labeling. Nat. Methods 17, 609–613. 10.1038/s41592-020-0823-y. 32424271 PMC8056749

[33] Lai HM, Tang Y, Lau ZYH, Campbell RAA, Yau JCN, Chan CCY, Chan DCW, Wong TY, Wong HKT, Yan LYC, (2022). Antibody stabilization for thermally accelerated deep immunostaining. Nat. Methods 19, 1137–1146. 10.1038/s41592-022-01569-1. 36050489 PMC9467915

[34] Susaki EA, Shimizu C, Kuno A, Tainaka K, Li X, Nishi K, Morishima K, Ono H, Ode KL, Saeki Y, (2020). Versatile whole-organ/body staining and imaging based on electrolyte-gel properties of biological tissues. Nat. Commun. 11, 1982. 10.1038/s41467-020-15906-5. 32341345 PMC7184626

[35] Zhao S, Todorov MI, Cai R, -Maskari RAI, Steinke H, Kemter E, Mai H, Rong Z, Warmer M, Stanic K, (2020). Cellular and Molecular Probing of Intact Human Organs. Cell 180, 796–812.e19. 10.1016/j.cell.2020.01.030. 32059778 PMC7557154

[36] Choi SW, Guan W, and Chung K (2021). Basic principles of hydrogel-based tissue transformation technologies and their applications. Cell 184, 4115–4136. 10.1016/j.cell.2021.07.009. 34358468 PMC8372535

[37] Yau CN, Lai HM, Lee K, Kwok AJ, Huang J, and Ko H (2023). Principles of deep immunohistochemistry for 3D histology. Cell Rep. Methods 3, 100458. 10.1016/j.crmeth.2023.100458. PMC10261851 37323568

[38] Richardson DS, Guan W, Matsumoto K, Pan C, Chung K, Ertürk A, Ueda HR, and Lichtman JW (2021). Tissue Clearing. Nat. Rev. Methods Primers 1, 84. 10.1038/s43586-021-00080-9. 35128463 PMC8815095

[39] Ueda HR, Ertürk A, Chung K, Gradinaru V, Chédotal A, Tomancak P, and Keller PJ (2020). Tissue clearing and its applications in neuroscience. Nat. Rev. Neurosci. 21, 61–79. 10.1038/s41583-019-0250-1. 31896771 PMC8121164

[40] Dejaegere A, Choulier L, Lafont V, De Genst E, and Altschuh D (2005). Variations in Antigen-Antibody Association Kinetics as a Function of pH and Salt Concentration: A QSAR and Molecular Modeling Study. Biochemistry 44, 14409–14418. 10.1021/bi050986v. 16262241

[41] Cann JR, and Clark EW (1956). Kinetics of the Antigen-Antibody Reaction. Effect of Salt Concentration and pH on the Rate of Neutralization of Bacteriophage by Purified Fractions of Specific Antiserum1a. J. Am. Chem. Soc. 78, 3627–3631. 10.1021/ja01596a020.

[42] Himo F, Lovell T, Hilgraf R, Rostovtsev VV, Noodleman L, Sharpless KB, and Fokin VV (2005). Copper(I)-catalyzed synthesis of azoles. DFT study predicts unprecedented reactivity and intermediates. J. Am. Chem. Soc. 127, 210–216. 10.1021/ja0471525. 15631470

[43] Krysiak JM, Kreuzer J, Macheroux P, Hermetter A, Sieber SA, and Breinbauer R (2012). Activity-based probes for studying the activity of flavin-dependent oxidases and for the protein target profiling of monoamine oxidase inhibitors. Angew. Chem. Int. Ed. Engl. 51, 7035–7040. 10.1002/anie.201201955. 22689512 PMC3470703

[44] Niphakis MJ, Johnson DS, Ballard TE, Stiff C, and Cravatt BF (2012). O-hydroxyacetamide carbamates as a highly potent and selective class of endocannabinoid hydrolase inhibitors. ACS Chem. Neurosci. 3, 418–426. 10.1021/cn200089j. 22860211 PMC3382460

[45] Festa RA, and Thiele DJ (2011). Copper: an essential metal in biology. Curr. Biol. 21, R877–R883. 10.1016/j.cub.2011.09.040. 22075424 PMC3718004

[46] Inesi G (2017). Molecular features of copper binding proteins involved in copper homeostasis. IUBMB Life 69, 211–217. 10.1002/iub.1590. 27896900

[47] Andreini C, Banci L, Bertini I, and Rosato A (2008). Occurrence of copper proteins through the three domains of life: a bioinformatic approach. J. Proteome Res. 7, 209–216. 10.1021/pr070480u. 17988086

[48] Selden CR, Schilling K, Godfrey L, and Yee N (2024). Metal-binding amino acid ligands commonly found in metalloproteins differentially fractionate copper isotopes. Sci. Rep. 14, 1902. 10.1038/s41598-024-52091-7. 38253574 PMC11229503

[49] Li S, Cai H, He J, Chen H, Lam S, Cai T, Zhu Z, Bark SJ, and Cai C (2016). Extent of the Oxidative Side Reactions to Peptides and Proteins During the CuAAC Reaction. Bioconjug. Chem. 27, 2315–2322. 10.1021/acs.bioconjchem.6b00267. 27583984

[50] Abel GR Jr., Calabrese ZA, Ayco J, Hein JE, and Ye T (2016). Measuring and Suppressing the Oxidative Damage to DNA During Cu(I)-Catalyzed Azide-Alkyne Cycloaddition. Bioconjug. Chem. 27, 698–704. 10.1021/acs.bioconjchem.5b00665. 26829457

[51] Wiest A, and Kielkowski P (2024). Cu-Catalyzed Azide-Alkyne-Thiol Reaction Forms Ubiquitous Background in Chemical Proteomic Studies. J. Am. Chem. Soc. 146, 2151–2159. 10.1021/jacs.3c11780. 38214237 PMC10811670

[52] Attarpour A, Osmann J, Rinaldi A, Qi T, Lal N, Patel S, Rozak M, Yu F, Cho N, Squair J, (2025). A deep learning pipeline for three-dimensional brain-wide mapping of local neuronal ensembles in teravoxel light-sheet microscopy. Nat. Methods 22, 600–611. 10.1038/s41592-024-02583-1. 39870865 PMC11903318

[53] Wang Q, Ding S-L, Li Y, Royall J, Feng D, Lesnar P, Graddis N, Naeemi M, Facer B, Ho A, (2020). The Allen mouse brain common coordinate framework: a 3D reference atlas. Cell 181, 936–953.e20. 10.1016/j.cell.2020.04.007. 32386544 PMC8152789

[54] Boike L, Henning NJ, and Nomura DK (2022). Advances in covalent drug discovery. Nat. Rev. Drug Discov. 21, 881–898. 10.1038/s41573-022-00542-z. 36008483 PMC9403961

[55] Ghosh AK, Samanta I, Mondal A, and Liu WR (2019). Covalent Inhibition in Drug Discovery. ChemMedChem 14, 889–906. 10.1002/cmdc.201900107. 30816012 PMC6816337

[56] Miller VA, Hirsh V, Cadranel J, Chen Y-M, Park K, Kim S-W, Zhou C, Su W-C, Wang M, Sun Y, (2012). Afatinib versus placebo for patients with advanced, metastatic non-small-cell lung cancer after failure of erlotinib, gefitinib, or both, and one or two lines of chemotherapy (LUX-Lung 1): a phase 2b/3 randomised trial. Lancet Oncol. 13, 528–538. 10.1016/s1470-2045(12)70087-6. 22452896

[57] Sequist LV, Yang JCH, Yamamoto N, O’Byrne K, Hirsh V, Mok T, Geater SL, Orlov S, Tsai CM, Boyer M, (2013). Phase III study of afatinib or cisplatin plus pemetrexed in patients with metastatic lung adenocarcinoma with EGFR mutations. J. Clin. Oncol. 31, 3327–3334. 10.1200/jco.2012.44.2806. 23816960

[58] Byrd JC, Furman RR, Coutre SE, Flinn IW, Burger JA, Blum KA, Grant B, Sharman JP, Coleman M, Wierda WG, (2013). Targeting BTK with ibrutinib in relapsed chronic lymphocytic leukemia. N. Engl. J. Med. 369, 32–42. 10.1056/NEJMoa1215637. 23782158 PMC3772525

[59] Treon SP, Tripsas CK, Meid K, Warren D, Varma G, Green R, Argyropoulos KV, Yang G, Cao Y, Xu L, (2015). Ibrutinib in previously treated Waldenstrom’s macroglobulinemia. N. Engl. J. Med. 372, 1430–1440. 10.1056/NEJMoa1501548. 25853747

[60] Wang ML, Rule S, Martin P, Goy A, Auer R, Kahl BS, Jurczak W, Advani RH, Romaguera JE, Williams ME, (2013). Targeting BTK with ibrutinib in relapsed or refractory mantle-cell lymphoma. N. Engl. J. Med. 369, 507–516. 10.1056/NEJMoa1306220. 23782157 PMC4513941

[61] Yun S, Vincelette ND, Acharya U, and Abraham I (2017). Risk of Atrial Fibrillation and Bleeding Diathesis Associated With Ibrutinib Treatment: A Systematic Review and Pooled Analysis of Four Randomized Controlled Trials. Clin. Lymphoma Myeloma Leuk. 17, 31–37.e13. 10.1016/j.clml.2016.09.010. 27780690

[62] Lipsky AH, Farooqui MZH, Tian X, Martyr S, Cullinane AM, Nghiem K, Sun C, Valdez J, Niemann CU, Herman SEM, (2015). Incidence and risk factors of bleeding-related adverse events in patients with chronic lymphocytic leukemia treated with ibrutinib. Haematologica 100, 1571–1578. 10.3324/haematol.2015.126672. 26430171 PMC4666333

[63] Berglöf A, Hamasy A, Meinke S, Palma M, Krstic A, Månsson R, Kimby E, Österborg A, and Smith CI (2015). Targets for Ibrutinib Beyond B Cell Malignancies. Scand. J. Immunol. 82, 208–217. 10.1111/sji.12333. 26111359 PMC5347933

[64] Tuomi JM, Xenocostas A, and Jones DL (2018). Increased Susceptibility for Atrial and Ventricular Cardiac Arrhythmias in Mice Treated With a Single High Dose of Ibrutinib. Can. J. Cardiol. 34, 337–341. 10.1016/j.cjca.2017.12.001. 29475534

[65] Xiao L, Salem JE, Clauss S, Hanley A, Bapat A, Hulsmans M, Iwamoto Y, Wojtkiewicz G, Cetinbas M, Schloss MJ, (2020). Ibrutinib-Mediated Atrial Fibrillation Attributable to Inhibition of C-Terminal Src Kinase. Circulation 142, 2443–2455. 10.1161/CIRCULA-TIONAHA.120.049210. 33092403 PMC9661397

[66] McMullen JR, Boey EJH, Ooi JYY, Seymour JF, Keating MJ, and Tam CS (2014). Ibrutinib increases the risk of atrial fibrillation, potentially through inhibition of cardiac PI3K-Akt signaling. Blood 124, 3829–3830. 10.1182/blood-2014-10-604272. 25498454

[67] Honigberg LA, Smith AM, Sirisawad M, Verner E, Loury D, Chang B, Li S, Pan Z, Thamm DH, Miller RA, (2010). The Bruton tyrosine kinase inhibitor PCI-32765 blocks B-cell activation and is efficacious in models of autoimmune disease and B-cell malignancy. Proc. Natl. Acad. Sci. USA 107, 13075–13080. 10.1073/pnas.1004594107. 20615965 PMC2919935

[68] Lanning BR, Whitby LR, Dix MM, Douhan J, Gilbert AM, Hett EC, Johnson TO, Joslyn C, Kath JC, Niessen S, (2014). A road map to evaluate the proteome-wide selectivity of covalent kinase inhibitors. Nat. Chem. Biol. 10, 760–767. 10.1038/nchembio.1582. 25038787 PMC4138289

[69] Kaltenecker D, Al-Maskari R, Negwer M, Hoeher L, Kofler F, Zhao S, Todorov M, Rong Z, Paetzold JC, Wiestler B, (2024). Virtual reality-empowered deep-learning analysis of brain cells. Nat. Methods 21, 1306–1315. 10.1038/s41592-024-02245-2. 38649742 PMC11239522

[70] Center for Drug Evaluation and Research (2013). Application number: 201292Orig1s000. https://www.accessdata.fda.gov/drugsatfda_docs/nda/2013/201292Orig1s000PharmR.pdf.

[71] Center for Drug Evaluation and Research (2014). Application number: 205552Orig2s000. https://www.accessdata.fda.gov/drugsatfda_docs/nda/2013/205552Orig1s000PharmR.pdf.

[72] Karlsson M, Zhang C, Méar L, Zhong W, Digre A, Katona B, Sjöstedt E, Butler L, Odeberg J, Dusart P, (2021). A single–cell type transcriptomics map of human tissues. Sci. Adv. 7, eabh2169. 10.1126/sciadv.abh2169. PMC8318366 34321199

[73] Fleming MR, Xiao L, Jackson KD, Beckman JA, Barac A, and Moslehi JJ (2021). Vascular Impact of Cancer Therapies: The Case of BTK (Bruton Tyrosine Kinase) Inhibitors. Circ. Res. 128, 1973–1987. 10.1161/CIRCRESAHA.121.318259. 34110908 PMC10185355

[74] Tom Dieck S, Müller A, Nehring A, Hinz FI, Bartnik I, Schuman EM, and Dieterich DC (2012). Chapter 7. Metabolic labeling with noncanonical amino acids and visualization by chemoselective fluorescent tagging. Curr. Protoc. Cell Biol. Chapter, 7.11.1–7.11.29. 10.1002/0471143030.cb0711s56. PMC3736104 22968844

[75] Jao CY, and Salic A (2008). Exploring RNA transcription and turnover in vivo by using click chemistry. Proc. Natl. Acad. Sci. USA 105, 15779–15784. 10.1073/pnas.0808480105. 18840688 PMC2572917

[76] Nikić I, Kang JH, Girona GE, Aramburu IV, and Lemke EA (2015). Labeling proteins on live mammalian cells using click chemistry. Nat. Protoc. 10, 780–791. 10.1038/nprot.2015.045. 25906116

[77] El-Sagheer AH, and Brown T (2010). Click chemistry with DNA. Chem. Soc. Rev. 39, 1388–1405. 10.1039/b901971p. 20309492

[78] Soriano del Amo D, Wang W, Jiang H, Besanceney C, Yan AC, Levy M, Liu Y, Marlow FL, and Wu P (2010). Biocompatible copper(I) catalysts for in vivo imaging of glycans. J. Am. Chem. Soc. 132, 16893–16899. 10.1021/ja106553e. 21062072 PMC3021957

[79] Hulce JJ, Cognetta AB, Niphakis MJ, Tully SE, and Cravatt BF (2013). Proteome-wide mapping of cholesterol-interacting proteins in mammalian cells. Nat. Methods 10, 259–264. 10.1038/nmeth.2368. 23396283 PMC3601559

[80] Tamura I, Sakamoto DM, Yi B, Saito Y, Yamada N, Morimoto J, Takakusagi Y, Kuroda M, Kubota SI, Yatabe H, (2024). Click3D: Click reaction across deep tissues for whole-organ 3D fluorescence imaging. Sci. Adv. 10, eado8471. 10.1126/sciadv.ado8471. PMC466963 39018410

[81] Shi MY, Yao Y, Wang M, Yang Q, Ding L, Li R, Li Y, Huang H, Yang CY, Zhou Z, (2025). High-speed mapping of whole-mouse peripheral nerves at subcellular resolution. Cell 188, 3897–3915.e20. 10.1016/j.cell.2025.06.011. 40645171

[82] Xu F, Shen Y, Ding L, Yang CY, Tan H, Wang H, Zhu Q, Xu R, Wu F, Xiao Y, (2021). High-throughput mapping of a whole rhesus monkey brain at micrometer resolution. Nat. Biotechnol. 39, 1521–1528. 10.1038/s41587-021-00986-5. 34312500

[83] Chung K, Wallace J, Kim SY, Kalyanasundaram S, Andalman AS, Davidson TJ, Mirzabekov JJ, Zalocusky KA, Mattis J, Denisin AK, (2013). Structural and molecular interrogation of intact biological systems. Nature 497, 332–337. 10.1038/nature12107. 23575631 PMC4092167

[84] Tang P-F, Bao S-S, Xiao Z-X, Xie W-F, Wu X-M, Ge H-L, and Shao C-F (2023). A novel UHPLC‒MS/MS method for quantitative analysis of zanubrutinib in rat plasma: application to an in vivo interaction study between zanubrutinib and triazole antifungal. BMC Chem. 17, 107. 10.1186/s13065-023-01017-x. 37649082 PMC10469817

[85] Lu X, Liu S, Yang X, Han M, and Sun K (2019). Determination of tyrosine kinase inhibitor afatinib in rat plasma using LC–MS/MS and its application to in vivo pharmacokinetic studies of afatinib liposomes. J. Pharm. Biomed. Anal. 164, 181–186. 10.1016/j.jpba.2018.10.043. 30390560

[86] Qi Y, Yu T, Xu J, Wan P, Ma Y, Zhu J, Li Y, Gong H, Luo Q, and Zhu D (2019). FDISCO: Advanced solvent-based clearing method for imaging whole organs. Sci. Adv. 5, eaau8355. 10.1126/sciadv.aau8355. PMC6357753 30746463

[87] Park YG, Sohn CH, Chen R, McCue M, Yun DH, Drummond GT, Ku T, Evans NB, Oak HC, Trieu W, (2018). Protection of tissue physicochemical properties using polyfunctional crosslinkers. Nat. Biotechnol. 37, 73–83. 10.1038/nbt.4281. PMC6579717 30556815

[88] Goubran M, Leuze C, Hsueh B, Aswendt M, Ye L, Tian Q, Cheng MY, Crow A, Steinberg GK, McNab JA, (2019). Multimodal image registration and connectivity analysis for integration of connectomic data from microscopy to MRI. Nat. Commun. 10, 5504. 10.1038/s41467-019-13374-0. 31796741 PMC6890789

[89] Arzt M, Deschamps J, Schmied C, Pietzsch T, Schmidt D, Tomancak P, Haase R, and Jug F (2022). LABKIT: labeling and segmentation toolkit for big image data. Front. Comput. Sci. 4, 777728. 10.3389/fcomp.2022.777728.

[90] Schindelin J, Arganda-Carreras I, Frise E, Kaynig V, Longair M, Pietzsch T, Preibisch S, Rueden C, Saalfeld S, Schmid B, (2012). Fiji: an open-source platform for biological-image analysis. Nat. Methods 9, 676–682. 10.1038/nmeth.2019. 22743772 PMC3855844

[91] Kinga D, and Adam JB (2015). A Method for Stochastic Optimization. Preprint at arXiv. 10.48550/arXiv.1412.6980.

[92] Wagner T, and Lipinski H-G (2013). IJBlob: an ImageJ library for connected component analysis and shape analysis. J. Open Res. Softw. 1, e6.

[93] Waskom ML (2021). Seaborn: statistical data visualization. J. Open Source Softw. 6, 3021. 10.21105/joss.03021.

[94] Verstegen AMJ, Vanderhorst V, Gray PA, Zeidel ML, and Geerling JC (2017). Barrington’s nucleus: Neuroanatomic landscape of the mouse “pontine micturition center”. J. Comp. Neurol. 525, 2287–2309. 10.1002/cne.24215. 28340519 PMC5832452

