# Supplementary material for: Mapping cellular targets of covalent cancer drugs in the entire mammalian body
Source: Cell. Author manuscript; Available in PMC 2026 Feb 6. (PMC12875305; doi:10.1016/j.cell.2025.11.030)
Supplement: 1 [file NIHMS2137673-supplement-1.pdf]

CellPress
OPEN ACCESS

Supplemental figures

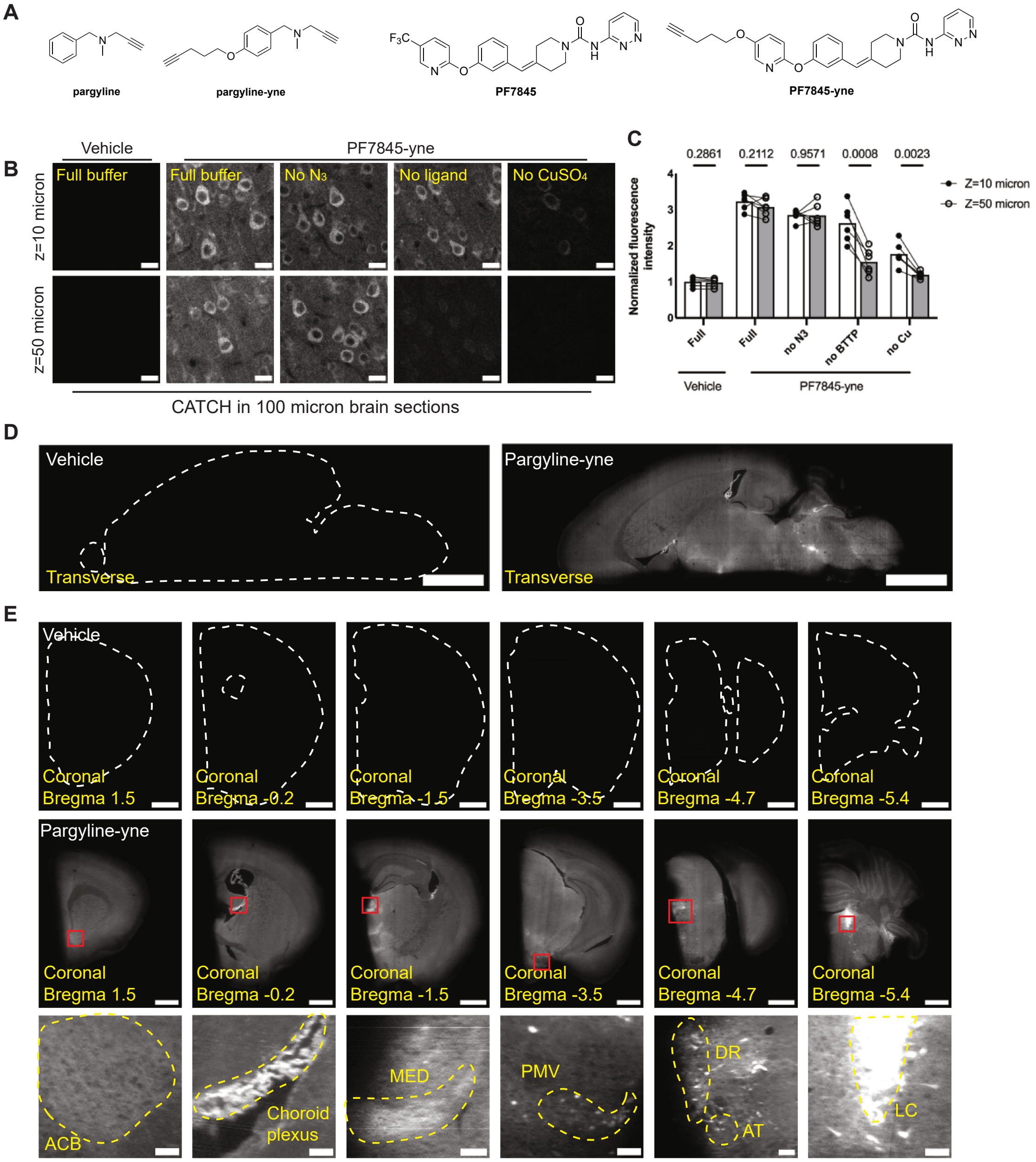

Figure S1. vCATCH development, related to Figure 1

(A) Chemical structure of the MAO-A/B inhibitor pargyline, FAAH inhibitor PF7845, and their alkyne analogs used in the study. Both analogs have been verified in previous CATCH and ABPP studies.

(B) CATCH in 100-micron vehicle or PF7845-yne-treated (1 mg/kg, 1 h i.p.) brain sections. Individual components (Cu, ligand, N_3) were removed during the click reaction incubation. Representative images were taken at the primary somatosensory cortex (S1, imaging depth Z = 10 and 50 microns).

(C) Quantification of CATCH intensity in (B). Six tissue sections (one FOV per section) were used for each condition. Intensity at Z = 10 or 50 microns was measured and then normalized to the average vehicle labeling intensity at Z = 10 or 50 microns, respectively. Paired *t* test in each condition. *p* values are plotted in the graph. *n* = 6 tissue sections for each condition.

(D) Transverse 2D views of hemispheres in Figure 1E.

(legend continued on next page)

(E) Coronal 2D views of hemispheres in Figure 1E. Zoomed-in views showing pargyline-yne-enriched structures across the whole brain.

Statistics determined by two-tailed paired *t* test in (C). Dashed lines indicate tissue boundary. Scale bars: 20 μm (B), 2,000 μm (D), 1,000 μm (E, coronal view), and 100 μm (E, zoom-in view).

ACB, nucleus accumbens; MED, medial group of the dorsal thalamus; PMV, ventral premammillary nucleus; DR, dorsal nucleus raphe; AT, anterior tegmental nucleus; LC, locus coeruleus.

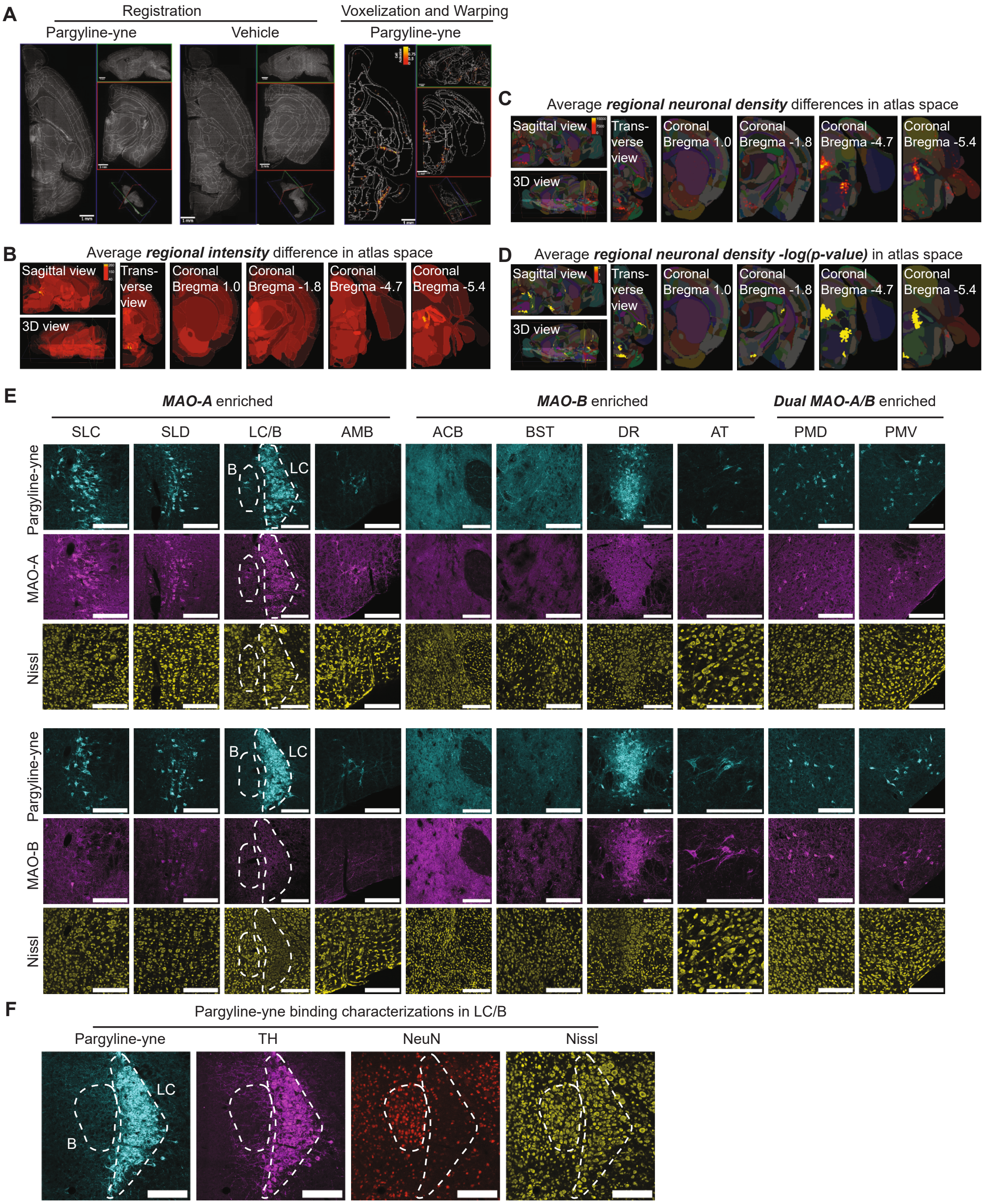

Figure S2. ACE analysis and validation, related to Figure 2

(A) Evaluation of ACE registration and warping algorithms. Lightsheet data were registered to the ABA using ACE. Left and right panels show data overlaid on labels for two subjects in each group. Segmentation maps from ACE were voxelized and warped to ABA 25-μm resolution. Panels show a randomly selected subject from the pargyline group.

(B) Atlas plot of the average intensity difference of vCATCH labeling intensity of vehicle vs. pargyline-yne-treated samples. The greatest labeling intensity elevation is denoted in yellow.

(C) Atlas plot of the average segmented neuronal density (number of cells per mm^3) of vehicle vs. pargyline-yne-treated samples. The greatest density elevation is denoted in yellow.

(D) Atlas plot of the average [−log(p value)] of segmented neuronal density in vehicle vs. pargyline-yne-treated samples. The greatest statistical significance is denoted in yellow.

(legend continued on next page)

(E) MAO-A and -B immunostaining in pargyline-yne-positive regions.
(F) Histology validation of pargyline-yne binding in the LC. The boundary of LC and B is identified by neuronal nuclei (NeuN) and tyrosine hydroxylase (TH) staining using a published protocol.[94] Pargyline-yne binding is restricted in LC, suggesting that "B" was likely a false positive from ACE due to its small size.
Scale bars: 200 μm (E and F).

CellPress
OPEN ACCESS

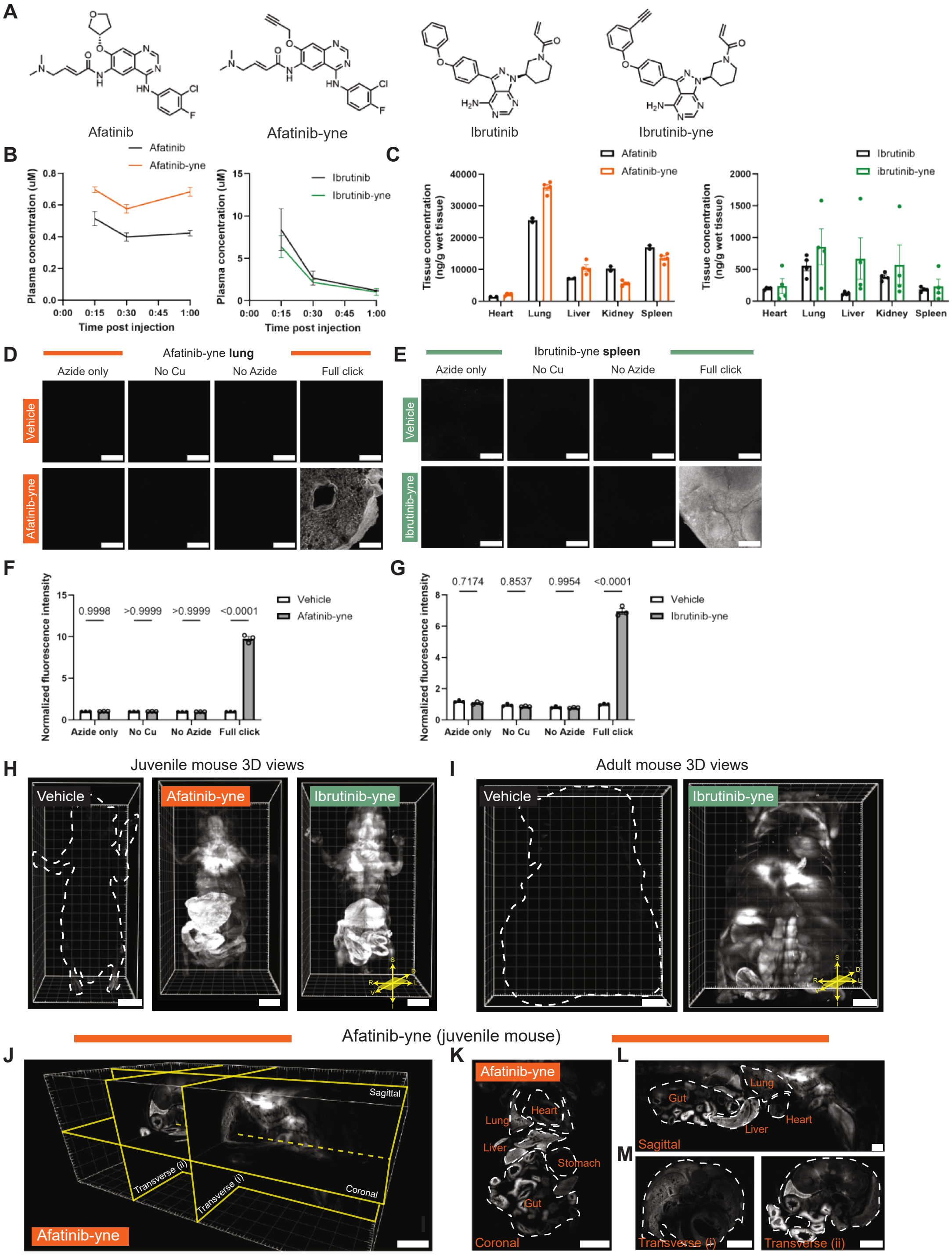

Figure S3. Whole-body vCATCH visualization of TKI binding, related to Figure 3

(A) Chemical structures of afatinib, ibrutinib, and their alkyne analogs.

(B) PK studies of plasma drug concentrations. Plasma samples were taken 15, 30, and 60 min after drug injection (10 mg/kg). Two parental afatinib-60 min samples were excluded as outliers. $n = 4$ mice for all time points except parental afatinib at 60 min ($n = 2$ mice).

(C) PK studies of tissue drug concentrations at 60 min post injection (10 mg/kg). $n = 4$ mice for afatinib-yne, ibrutinib, and ibrutinib-yne; $n = 2$ mice for afatinib.

(D and E) Click reaction specificity validation in afatinib-yne-treated lung (D) and ibrutinib-yne-treated spleen (E).

(F and G) Quantifications of click labeling intensity in afatinib-yne-treated lung (F) and ibrutinib-yne-treated spleen (G). Intensity normalized to vehicle full click condition. Three independent tissues were used for quantification. Two-way ANOVA with Šidák multiple comparisons test.

(legend continued on next page)

(H) Representative 3D lightsheet image volume of whole-body drug distribution (10 mg/kg, 1 h, i.p.) in juvenile vehicle, afatinib-yne, and ibrutinib-yne mice. D, dorsal; V, ventral; S, superior; I, inferior; R, right; L, left.
(I) Adult whole torso 3D overview of mice treated with vehicle and ibrutinib-yne (10 mg/kg, 1 h, i.p.).
(J) Afatinib-yne distribution overviews with digital plane slicing in 3D volume image.
(K–M) Coronal (K), sagittal (L), and transverse (M) views of mice injected with afatinib-yne as shown in (J).
Data are plotted as mean ± SEM. Dashed lines indicate tissue boundary. Scale bars: 200 μm (D and E), 4,000 μm (H–K), and 2,000 μm (L and M).

CellPress
OPEN ACCESS

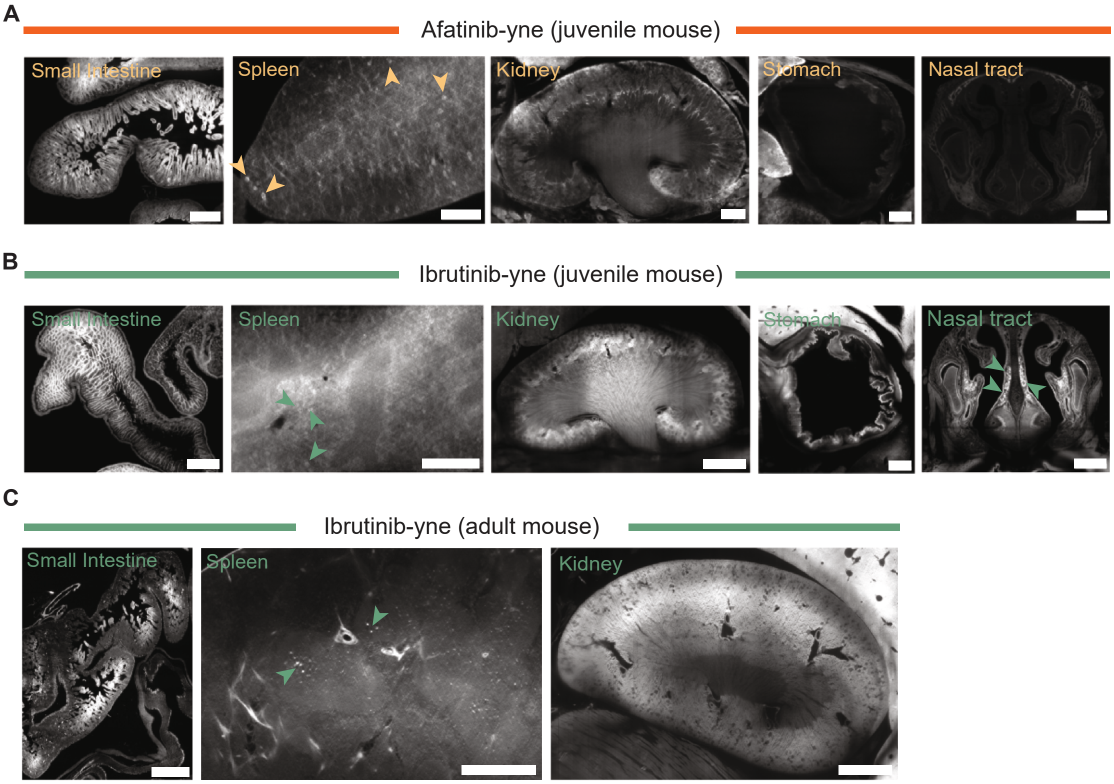

Figure S4. Organ-level TKI binding, related to Figure 4

(A) Representative organ views from afatinib-yne-treated mice. Afatinib-yne enrichment was observed in small intestine villi, spleen central arterioles (orange arrows), and kidney cortex. No significant drug enrichment was observed in the stomach lining or nasal tract. Scale bars: small intestine, kidney, stomach, and nasal tract, 500 μm; spleen, 250 μm.

(B) Representative organ views from ibrutinib-yne-treated mice. Ibrutinib-yne enrichment was observed in small intestine villi, spleen central arterioles (green arrows), kidney, and inner stomach lining. Scale bars: kidney and stomach, 500 μm; small intestine and nasal tract, 300 μm; spleen, 200 μm.

(C) Representative organ views from ibrutinib-yne-treated adult mice. Ibrutinib-yne enrichment was observed in small intestine villi, spleen central arterioles (green arrows), and kidney. Scale bars: small intestine, 1,000 μm; spleen, 500 μm; kidney, 1,500 μm.

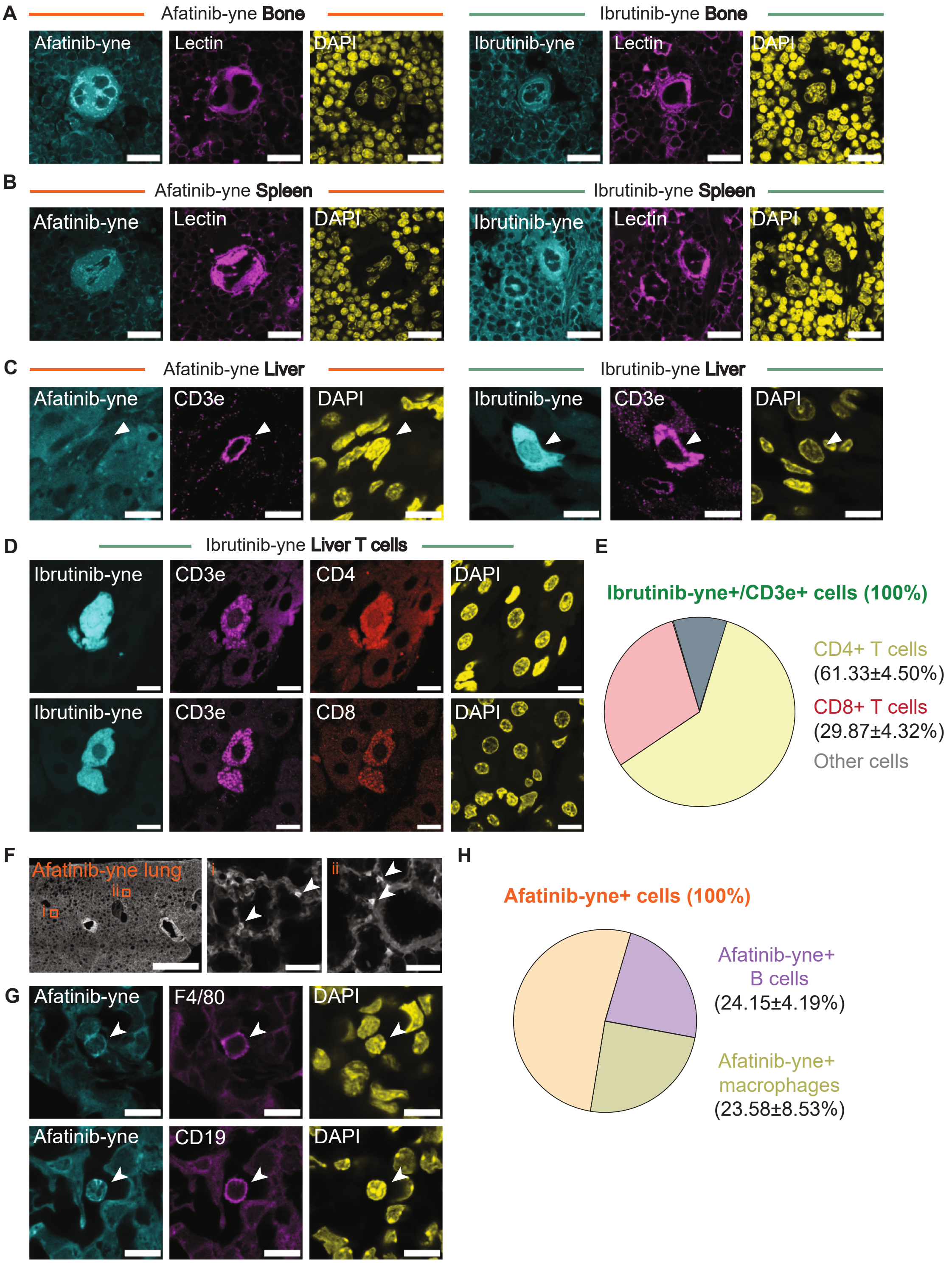

Figure S5. TKI-positive cell-type characterization, related to Figure 5

(A and B) Lectin staining in bone (A) and spleen (B) sections after CATCH labeling. Representative images showing lectin overlaps TKI-enriched labeling structures.

(C) High-resolution afatinib-yne and ibrutinib T cell characterization in the liver. Representative images show CD3e+ cells that are afatinib− (left) and ibrutinib+ (right). Arrows indicate CD3e+ cells.

(D) Cell-type characterizations of ibrutinib-yne-positive liver T cells. Samples are co-stained for helper T cells (CD4) and cytotoxic T cells (CD8).

(legend continued on next page)

(E) Quantifications of ibrutinib-yne-positive T cell subtypes in the liver. n = 4 biological replicates, and 11–17 FOV were acquired for each mouse.
(F) Global characterization of afatinib-yne binding in the lung. Zoom-in views showing discrete afatinib-yne-enriched structures scattered throughout the lung.
(G) High-resolution afatinib-yne cell-type characterization in the lung. Representative images showing afatinib-yne-enriched F4/80-positive macrophages (top) and CD19-positive B cells (bottom).
(H) Cell-type characterization of afatinib-yne-enriched cells in the lung. n = 4 biological replicates, and 5–6 FOV were acquired for each mouse.
All values are shown as mean ± SEM. Detailed cell-type quantification is in Table S1. Scale bars: 20 μm (A and B), 10 μm (C, D, and G), 1,000 μm (F, global view), and 50 μm (F, zoomed-in view).
